# Receptor Ligand-Free Mesoporous Silica Nanoparticles: A Streamlined Strategy for Targeted Drug Delivery across the Blood–Brain Barrier

**DOI:** 10.1021/acsnano.3c08993

**Published:** 2024-05-08

**Authors:** Zih-An Chen, Cheng-Hsun Wu, Si-Han Wu, Chiung-Yin Huang, Chung-Yuan Mou, Kuo-Chen Wei, Yun Yen, I-Ting Chien, Sabiha Runa, Yi-Ping Chen, Peilin Chen

**Affiliations:** † Department of Chemistry, National Taiwan University, Taipei 10617, Taiwan; ‡ Graduate Institute of Nanomedicine and Medical Engineering, Taipei Medical University, Taipei 11031, Taiwan; § Research Center for Applied Sciences, Academia Sinica, Taipei 11529, Taiwan; ∥ Nano Targeting & Therapy Biopharma Inc., Taipei 10087, Taiwan; ⊥ International Ph.D. Program in Biomedical Engineering, Taipei Medical University, Taipei 11031, Taiwan; # Neuroscience Research Center, Chang Gung Memorial Hospital, Taoyuan 33305, Taiwan; ¶ Department of Neurosurgery, Chang Gung Memorial Hospital, Taoyuan 33305, Taiwan; ▲ School of Medicine, Chang Gung University, Taoyuan 33302, Taiwan; ● Department of Neurosurgery, New Taipei Municipal TuCheng Hospital, New Taipei City 23652, Taiwan; △ Center for Cancer Translational Research, Tzu Chi University, Hualien 970374, Taiwan; ▽ Cancer Center, Taipei Municipal WanFang Hospital, Taipei 116081, Taiwan; ▼ SRS Medical Communications, LLC, Cleveland, Ohio 44124, United States

**Keywords:** mesoporous silica nanoparticles, brain tumor, blood−brain barrier, the enhanced permeability and retention effect, doxorubicin, protein corona

## Abstract

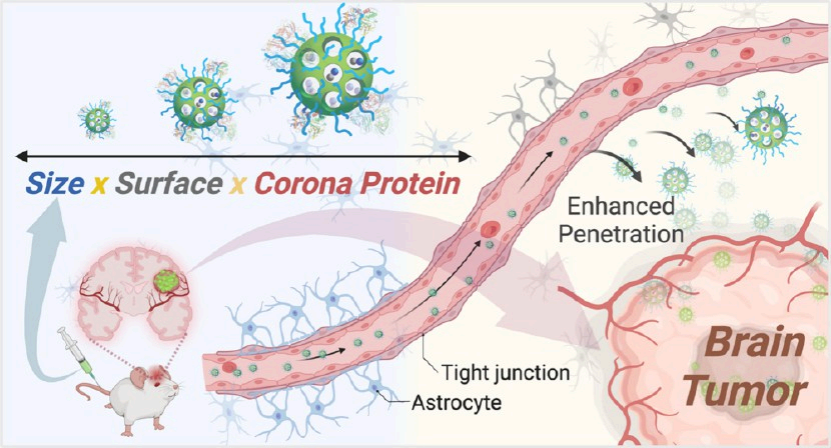

Mesoporous silica nanoparticles (MSNs) represent a promising avenue for targeted brain tumor therapy. However, the blood–brain barrier (BBB) often presents a formidable obstacle to efficient drug delivery. This study introduces a ligand-free PEGylated MSN variant (RMSN_25_-PEG-TA) with a 25 nm size and a slight positive charge, which exhibits superior BBB penetration. Utilizing two-photon imaging, RMSN_25_-PEG-TA particles remained in circulation for over 24 h, indicating significant traversal beyond the cerebrovascular realm. Importantly, DOX@RMSN_25_-PEG-TA, our MSN loaded with doxorubicin (DOX), harnessed the enhanced permeability and retention (EPR) effect to achieve a 6-fold increase in brain accumulation compared to free DOX. In vivo evaluations confirmed the potent inhibition of orthotopic glioma growth by DOX@RMSN_25_-PEG-TA, extending survival rates in spontaneous brain tumor models by over 28% and offering an improved biosafety profile. Advanced LC-MS/MS investigations unveiled a distinctive protein corona surrounding RMSN_25_-PEG-TA, suggesting proteins such as apolipoprotein E and albumin could play pivotal roles in enabling its BBB penetration. Our results underscore the potential of ligand-free MSNs in treating brain tumors, which supports the development of future drug–nanoparticle design paradigms.

## Introduction

Glioblastoma multiforme (GBM), characterized by its poor prognosis and a 5-year survival rate below 5%, presents a significant therapeutic challenge due to the blood-brain barrier (BBB) and blood-brain tumor barrier (BBTB) impeding effective drug delivery.^1−4^ Current systemic chemotherapies, including Temozolomide (TMZ), demonstrate limited efficacy largely due to these barriers and inherent drug resistance.^5,6^ Enhancing drug lipophilicity to facilitate BBB crossing has been explored,^7,8^ but this often results in poor penetration and systemic toxicity. To address these challenges, recent advances in nanomedicine have led to the development of nanodrug delivery systems (NDDS), offering strategies for therapeutic delivery to brain tumors.^9−15^

Crucially, in the realm of cancer nanomedicine, the size of nanoparticles (NPs) is a key factor for effective penetration into brain tumors. Smaller NPs, approximately 25 nm in diameter, are more adept at infiltrating the central hypoxic zones of tumors, a characteristic particularly vital in GBM.^16−18^ This penetration is supported by trans-endothelial pathways, an efficient route for nanoparticle movement into tumors.^19,20^ Concurrently, the enhanced permeability and retention (EPR) effect, traditionally credited for improved tumor accumulation, also plays a role in the preferential localization of nanoparticles within tumor tissue.^21^

Amidst these developments, specific drugs like Doxorubicin (DOX) have been scrutinized for their effectiveness against glioma cell lines and tumor models. Despite DOX’s potency, its activity is hampered by low lipid solubility and an inability to cross the BBB. A phase II clinical trial of TMZ combined with polyethylene glycolylated (PEGylated) liposomal DOX in patients with GBM reported good tolerability. However, the trial did not meet its primary end points of significantly improved 6-month progression free survival (6PFS) and overall survival (OS).^22,23^ This highlights an unmet need for targeted therapies in conjunction with standard treatments to improve outcomes for patients with GBM.

In addressing the challenges of drug delivery for brain tumors, mesoporous silica nanoparticles (MSNs) have emerged as a promising solution. Known for their biocompatibility, customizable properties, and high drug-loading capacities, MSNs are ideally suited for targeted and controlled drug delivery.^24−26^ Their synthesis can be meticulously tailored to achieve specific particle sizes and pore surface functionalities, enabling the encapsulation of a variety of drugs within the MSNs.^27^ A significant area of research on MSNs involves the application of PEGylation strategies. These strategies explore the impact of PEG’s molecular weight and density on factors such as blood circulation, degradation, hemolysis, and mucosal penetration.^25,26^ Crucially, efforts have been made to integrate PEGylation with active targeting ligands, enhancing the ability of MSNs to specifically target brain tumors.^28,29^ This dual approach of customizing both the internal (pores) and external surfaces of MSNs allows for precise control over drug loading and the pharmacokinetics (PKs) of the delivered payloads. While MSNs share a similar concept of nanocarriers with other NPs, the “distinctive mesoporous scaffolds,” “facades,” and “interior designs” can be fine-tuned, offering specialized utilities.^30^

In the broader context of NDDS, there has been a concerted effort to enhance the crossing of the BBB by functionalizing nanoparticles with specific ligands. These ligands target proteins associated with the brain’s microvasculature, facilitating receptor-mediated transcytosis.^31^ A notable example includes glutathione (GSH), a shuttle peptide conjugated onto NPs to aid BBB penetration. Previous reports have discussed GSH-conjugated magnetic NPs for magnetic resonance imaging (MRI) of brain tumors^32^ and GSH PEGylated liposomes-DOX for therapeutics in mice with experimental glioblastomas.^33^ Another popular peptide for overcoming the BBB is integrin-targeting arginine-glycine-aspartic acid (RGD)-based peptides, which have been employed on the surface of Cornell dots,^34^ liposomes^35^ and protein NPs^36^ with varying degrees of success in shrinking glioblastomas in mouse models. However, such strategies have significant brain-specificity limitations, as these target proteins are not exclusively expressed by the brain’s vasculature. Other findings reported design of a brain tumor–homing tetra-peptide.^37^ However, NPs with surface-attached peptide ligands might not work as intended due to the complex and uncertain protein corona adsorbed onto their surface.^38^ Also, the complex conjugation procedure and drug encapsulation processes complicate the scale-up manufacturing of the final drugs.

Another approach involves exploiting the natural protein corona, particularly focusing on apolipoproteins such as Apo-E, known to facilitate BBB penetration.^39^ ApoE, serving as the principal cholesterol carrier in the brain, plays a crucial role in lipid transport and neuronal uptake.^40^ Studies have demonstrated that ApoE functionalization on NPs significantly enhances their BBB crossing capabilities.^41,42^

Despite complexities in traditional methods, our research introduces a receptor ligand-free approach utilizing MSNs for GBM treatment. This strategy emphasizes targeted drug delivery across the BBB, achieved through tailed surface modifications of the MSNs. The specialized engineering of our MSNs includes: (a) precise control over size and surface properties to optimize delivery, (b) design that leverages the EPR effect for effective tumor targeting, (c) capability to overcome the BBB, and (d) utilization of apolipoproteins in blood plasma for improved targeting of the BBB.^23^ In addition, we show the complete ablation of xenograft brain tumors and extension of the life of mice in a spontaneous tumor model upon intravenous delivery of DOX@MSNs.

## Results

### Synthesis and Characterization of Functionalized PEGylated Mesoporous Silica Nanoparticles for BBB Penetration

**Figure 1:**
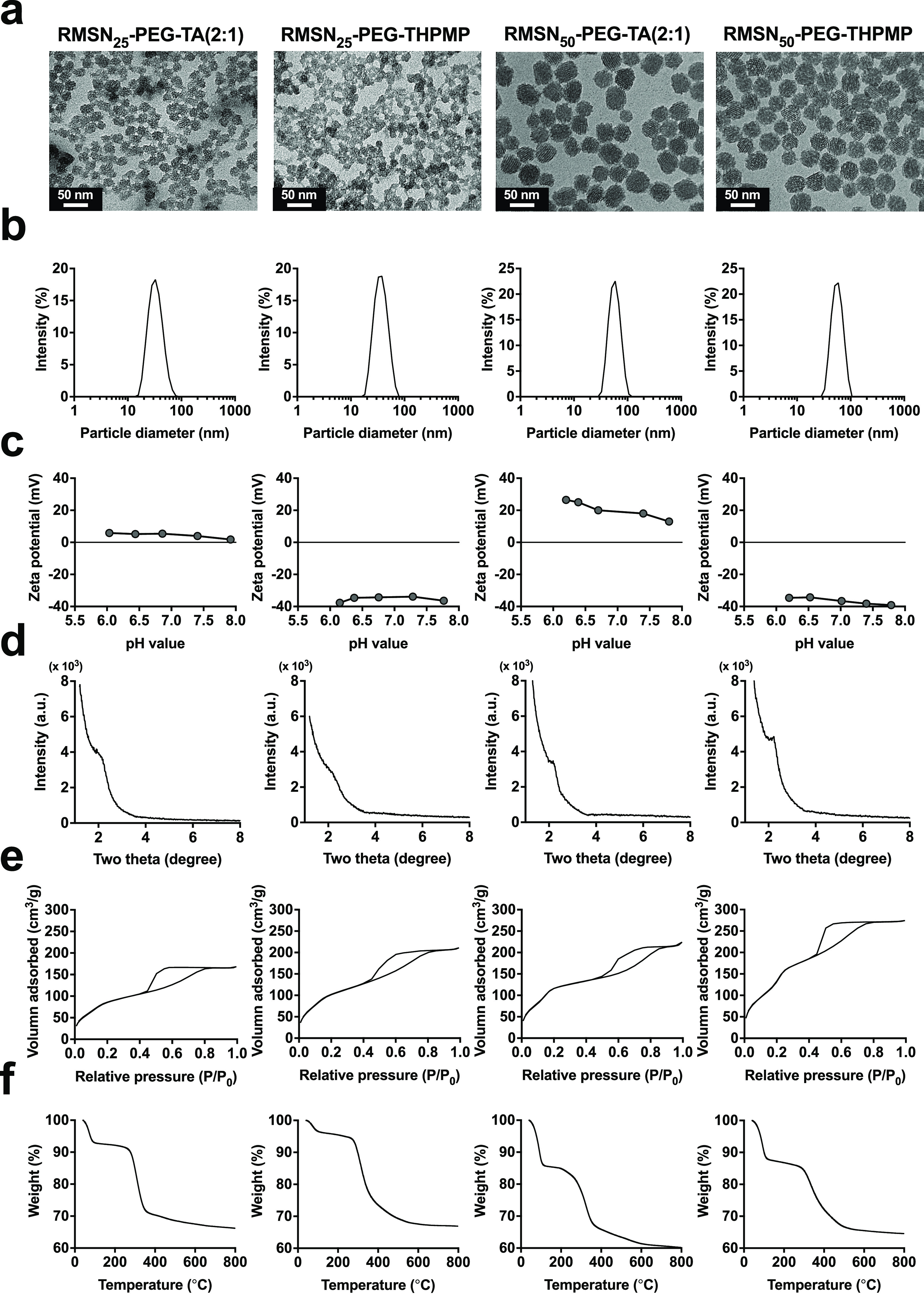
Physical characterizations of mesoporous silica nanoparticles (MSNs). (a) TEM images, (b) hydrodynamic diameter, (c) zeta potentials, (d) X-ray diffraction patterns, (e) nitrogen sorption isotherms, and (f) thermogravimetric analyses from left to right of RMSN_25_–PEG-TA(2:1), RMSN_25_-PEG-THPMP, RMSN_50_-PEG-TA(2:1), and RMSN_50_-PEG-THPMP. Detailed results are described in Tables S1 and S2 (in Supporting Information).

To investigate the influence of the NP size and surface charge on BBB penetration, we synthesized four types of functionalized PEGylated MSNs (MSN-PEG). MSN-PEG were functionalized with a red fluorescent dye (rhodamine isothiocyanate (RITC)) to form RMSN-PEG for visualizing the distribution of NPs under a fluorescence microscopy. RMSN-PEG with diameters of 25 and 50 nm were modified with quaternary ammonium groups to confer a positive charge (TA-silane) or methyl phosphonate groups to confer a negative charge (THPMP-silane). Specifically, when the molar ratio of PEG groups to TA groups was 2:1, they were named RMSN_25_–PEG-TA(2:1) and RMSN_50_-PEG-TA(2:1). Those with the methyl phosphonate group were named RMSN_25_–PEG-THPMP and RMSN_50_-PEG-THPMP, respectively. All MSNs were subjected to transmission electron microscopic (TEM) measurements, shown in Figure 1a. Respective average TEM sizes of RMSN_25_–PEG-TA(2:1), RMSN_25_–PEG-THPMP, RMSN_50_-PEG-TA(2:1), and RMSN_50_-PEG-THPMP were 22.2 ± 2.9 nm, 21.0 ± 3.3 nm, 48.1 ± 4.9 nm, and 46.7 ± 4.7 nm, respectively (Table S1). All RMSNs were uniform in size, with small standard deviations ranging from 10% to 15%. The resulting RMSN_25_–PEG-TA(2:1), RMSN_50_-PEG-TA(2:1), RMSN_25_–PEG-THPMP, and RMSN_50_-PEG-THPMP had hydrodynamic diameters (Z-average) of 32.2, 55.7, 34.6, and 54.8 nm, respectively, as determined by dynamical light scattering (DLS), indicating little aggregation in solution. Monodispersed size distributions, as determined by single-peak DLS histogram distributions (as percent intensities), and a polydispersity index (PDI) values <0.1 were obtained (Figure 1b, and Table S1). Surface charges of RMSN_25_–PEG-TA(2:1) and RMSN_50_-PEG-TA(2:1) according to ζ-potential analyses were close to neutral and positively charged (+4.0 mV and +18 mV at pH 7.4, respectively) due to the positively charged groups from TA-silane on the surface of the MSNs. Also, the surface charges of RMSN_25_–PEG-THPMP and RMSN_50_-PEG-THPMP were negative (−33.8 mV and −38.2 mV at pH 7.4, respectively) because of the phosphonate groups from THPMP-silane (Figure 1c and Table S1). X-ray diffraction (XRD) analyses showed a broad (100) peak which suggested short-range ordering of structure of the MSNs (Figure 1d). The interplanar spacing values, calculated from the Bragg peak position, for these MSNs were similar at nearly 4 nm (Table S1). Figure 1e shows the N_2_ adsorption–desorption isotherms of MSNs categorized as type IV behaviors with an obvious hysteresis loop associated with the mesoporous materials’ adsorption–desorption. All four types of MSNs showed surface areas of 319 to 590 m^2^ g as calculated by the Brunauer-Emmet-Teller (BET) equation and pore size distribution curves with a pore size of about 1.5 nm by the Barrett–Joyner–Halenda (BJH) method (Table S1). Results of thermogravimetric analyses (TGA) are shown in Figure 1f, and three steps of weight loss from 40 to 800 °C are summarized in Table S2. The first step of weight loss (expressed as a percentage of the initial weight) in the range of 40–250 °C for all samples was due to the loss of adsorbed water. The second step, beginning at 250 °C, was due to the decomposition of functional groups on the MSNs. Roughly one-quarter weight losses were observed for RMSN_25_–PEG-TA(2:1) and RMSN_25_–PEG-THPMP at 250 to 500 °C (22.9% and 25.4%, respectively); weight losses for both RMSN_50_-PEG-TA(2:1) and RMSN_50_-PEG-THPMP at 250 to 500 °C were 19.4%.

### *In Vitro* and *In Vivo* Studies of BBB Penetration and Blood Circulation of MSNs

**Figure 2:**
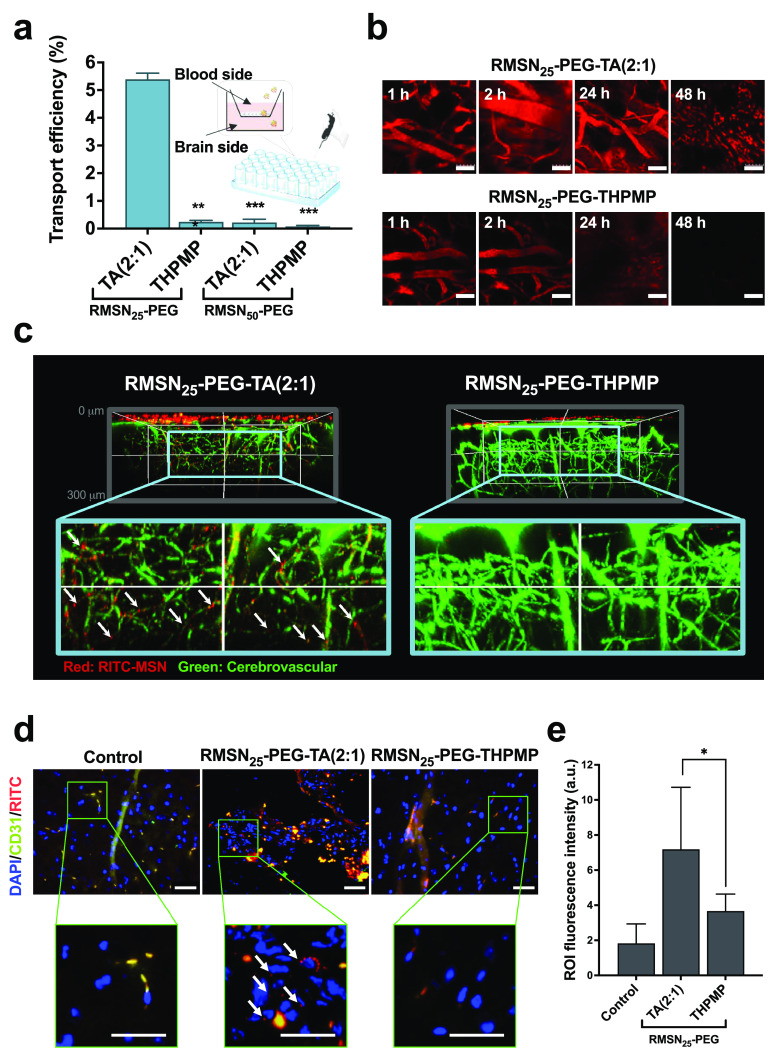
Penetration ability of RMSN_25_ as studied by an *in vitro* blood–brain barrier (BBB) model and two-photon *in vivo* images of mouse brains. (a) The transport efficiency of the *in vitro* BBB model incubated with 0.1 mg/mL of RMSN_25_-PEG-TA(2:1), RMSN_25_-PEG-THPMP, RMSN_50_-PEG-TA(2:1), or RMSN_50_-PEG-THPMP for 6 h and quantified by an ICP-OES analysis. (b) Multiphoton laser scanning microscopy imaged the circulation of RMSN_25_-PEG-TA(2:1) and RMSN_25_-PEG-THPMP inside the blood vessels. ICR mice were intravenously injected with nanoparticles at a dose of 200 mg/kg body weight, and the images from mouse ears were obtained within 1 to 48 h. Scale bar = 70 μm. (c) The same mice were anesthetized, and the procedure of a skull-removal craniotomy was then conducted. Images (at a depth up to 300 μm) of the mice cerebrum were observed by using multiphoton laser scanning microscopy with the cerebrovasculature (green signals) stained with dextran-conjugated FITC dye. The white arrowhead points to red signals of the RMSN_25_-PEG-TA(2:1) outside of the cerebrovasculature. (d) IF-stained images of a mouse cerebrum. Mice were sacrificed, and brain tissue sections were stained after perfusion. Red, green, and blue signals, respectively, represent RITC-conjugated MSNs, FAM Fluor 488-stained CD31 (cerebrovasculature), and DAPI-stained cell nuclei. The white arrowhead indicates that the red signal of the RMSN_25_-PEG-TA was not colocalized with green signals of blood vessels but present surrounding or nearby the cell nuclei. Scale bar = 40 μm. (e) Quantitative fluorescence image analysis based on intensities of regions of interest (ROIs) of three different regions was calculated by ImageJ software, shown in Figure S2. Data are presented as the mean ± SD (*n* = 3). *p < 0.05.

The *in vitro* and *in vivo* BBB penetration capabilities of MSNs were examined. Figure 2a is a schematic illustration of the *in vitro* BBB model, which was carried out using human cerebral endothelial cells cultured on a transwell inserted within a chamber to mimic the cell layer of the BBB. As a widely used electrical parameter, transepithelial/transendothelial electrical resistance (TEER) assesses the cellular barrier tightness of *in vitro* BBB transwell culture systems. All TEER values exceeded 100 Ω/cm^2^ for the four types of functionalized MSNs (0.1 mg/mL) for 6 h, indicating the BBB integrity (Figure S1a). For quantitative analysis of the transportation of NPs, the apical and basolateral media from the *in vitro* BBB model were collected, and the silica content was quantified by an inductive coupled plasma optical emission spectroscopic (ICP-OES) analysis (Figure 2a, and Table S3). The highest efficiency was observed with RMSN_25_–PEG-TA(2:1) at around 5.38% ± 0.2%. With a larger particle size, only 0.22% ± 0.11% of RMSN_50_-PEG-TA(2:1) could pass the cell layer. In addition, negatively charged MSNs (RMSN_25_–PEG-THPMP and RMSN_50_-PEG-THPMP) showed lower transport efficiencies of 0.24% ± 0.05% and 0.08% ± 0.03%, respectively, potentially as a result of their charge and size. These results strongly suggested that a small size (25 nm) and a slight positive charge were favorable properties for MSNs to cross this BBB model.

A long blood circulation time is one of the critical requirements for promoting the NPs to cross the BBB. To evaluate the circulation of MSNs in the blood, we first tracked their presence in the blood vessels of mice ears using multiphoton laser scanning microscopy (LSM) in Figure 2b. Considering the *in vitro* BBB model results, we focused on 25 nm MSNs for the remaining study. RMSN_25_–PEG-TA(2:1) and RMSN_25_–PEG-THPMP were injected intravenously into healthy mice at 200 mg/kg body weight (BW). The NP distribution was determined within 1 to 48 h by detecting RITC dyes conjugated onto the MSNs.

Not surprisingly, at 1 and 2 h after administering both MSNs, RITC fluorescent signals (red) were significantly observed inside the blood vessels, as shown in Figure 2b. At 24 h after the injection, RMSN_25_–PEG-TA(2:1) still showed persistent circulation in the bloodstream. In comparison, the fluorescence signals from RMSN_25_–PEG-THPMP had gradually disappeared from the blood vessels within 24 h. Both MSNs were lost from the circulation inside the blood vessels by 48 h after administration. Therefore, these results indicate that RMSN_25_–PEG-TA(2:1) retained a more-extended time circulation in the bloodstream and surface modification dramatically affected the behavior of NP circulation.

The same mice were anesthetized and underwent a skull-removal craniotomy to explore whether the MSNs had penetrated the BBB. Skulls of mice were partially cut open around the regions of interest (ROIs) and were observed by two-photon LSM to obtain deep-brain tissue imaging. To confirm whether MSNs had leaked from the blood vessels, images of the cerebrovasculature (green signals) were stained following an injection of dextran-conjugated FITC dye. As shown in Figure 2c, at a designated observational depth extending to 300 μm below the cortical surface, the red signs (indicated by the white arrowhead) of RMSN_25_–PEG-TA(2:1) were observed, and were prominently localized near the cerebrovascular structures of the cerebrum. In contrast, RMSN_25_–PEG-THPMP only appeared on the cortical surface, suggesting that they did not penetrate the BBB.

To further support the results obtained by the multiphoton microscopy, we investigated the destination of MSNs with immunofluorescent (IF) staining (Figure 2d). After fixation and paraffin embedding, the brain sections were stained with anticluster of differentiation (CD)-31 (green) and 4′,6-diamidino-4-phenylindole (DAPI) (blue) to respectively visualize the cerebrovasculature and cell nuclei. Fluorescence-merged images (orange) of RMSN_25_–PEG-TA(2:1) (red, RITC) overlapped with the distribution of blood vessels (green, anti-CD-31), revealing that some of the NPs were localized inside the cerebrovasculature. Interestingly, some RMSN_25_–PEG-TA(2:1) were present in the surrounding or nearby the cell nuclei (blue, DAPI) without colocalization with blood vessels (white arrowhead). In comparison, RMSN_25_–PEG-THPMP could not penetrate the BBB as shown in Figure 2c and 2d. These brain images demonstrated an enhancement of the satability of RMSN_25_–PEG-TA(2:1) to cross the BBB. The quantitative red fluorescence intensity (RITC) was measured in three different regions based on intensities of ROIs (Figure 2e and squares enclosed by green lines in Figure S2), indicating that the administration of RMSN_25_–PEG-TA(2:1) showed higher RITC signals compared with that of RMSN_25_–PEG-THPMP. Results suggest that RMSN_25_–PEG-TA(2:1) could successfully traverse the BBB, which is consistent with the results of multiphoton microscopy (Figure 2c).

To further investigate whether BBB permeability was achieved through barrier disruption or other fundamental mechanisms (e.g., transcytosis), distributions of three major proteins (CD31, zonula occludens (ZO)-1, and CD-140-b) associated with the BBB structure and RMSN_25_–PEG-TA(2:1) were imaged using confocal microscopy (Figure S3). U87MG-Luc tumor-bearing mice were sacrificed at 48 h postinjection with RMSN_25_–PEG-TA(2:1) at 200 mg/kg BW, followed by an IF staining analysis. CD31 proteins, also known as platelet endothelial cell adhesion molecule (PECAM)-1, were present in endothelial cells (ECs), which are a marker of blood vessels of the BBB.^43^ As shown in Figure S3, the red fluorescence signals from RMSN_25_–PEG-TA(2:1) were partially colocalized with green-labeled CD-31 of blood vessels (yellow arrowhead). Importantly, pronounced RMSN_25_–PEG-TA(2:1) signals were observed surrounding the nuclei stained by DAPI (blue) were far removed from the blood vessels (white arrowhead). Results demonstrated that RMSN_25_–PEG-TA(2:1) enabled the blood-brain tumor barrier (BBTB) penetration to achieve subsequent accumulation in the brain. Pericytes are cells tightly wrapped around the ECs, which provide the barrier structural support and are related to the transcellular mechanism.^44^ The middle image in Figure S3 shows that RMSN_25_–PEG-TA(2:1) (red, RITC) were partially colocalized with the pericytes (green, anti-CD-140b), clearly revealing that the transcellular diffusion mechanism (transcytosis) might have contributed to MSNs crossing the BBB (yellow arrowhead). More fundamental research is needed to understand the mechanism. In addition, a tight junctions (TJs) are a dynamic structures composed of membrane-associated cytoplasmic proteins, which are the main barrier to the paracellular diffusion of molecules and restricts the transportation of substances from the blood to the brain.^45^ Signals of both RMSN_25_–PEG-TA(2:1) (red, RITC) and TJ adhesion protein (green, anti-ZO-1) were observed (yellow arrowhead), as shown in Figure S3. Interestingly, RMSN_25_–PEG-TA(2:1) did not significantly colocalize with the TJ adhesion protein (ZO-1), suggesting the integrity of TJs in the areas of the brain tumors (white arrowhead). Thus, we reasoned that the transcytosis mechanism is the major route employed by RMSN_25_–PEG-TA(2:1) to cross the BBB. Taken together, *in vivo* confocal images demonstrated that RMSN_25_–PEG-TA(2:1) could pass the BBB and subsequently accumulate in the tumor of mouse brains through transcytosis, which is also consistent with the results of Figure 2d.

### Effect of Surface Functionalization of 25 nm MSNs on the Biodistribution and Tumor-Targeting Ability

**Figure 3:**
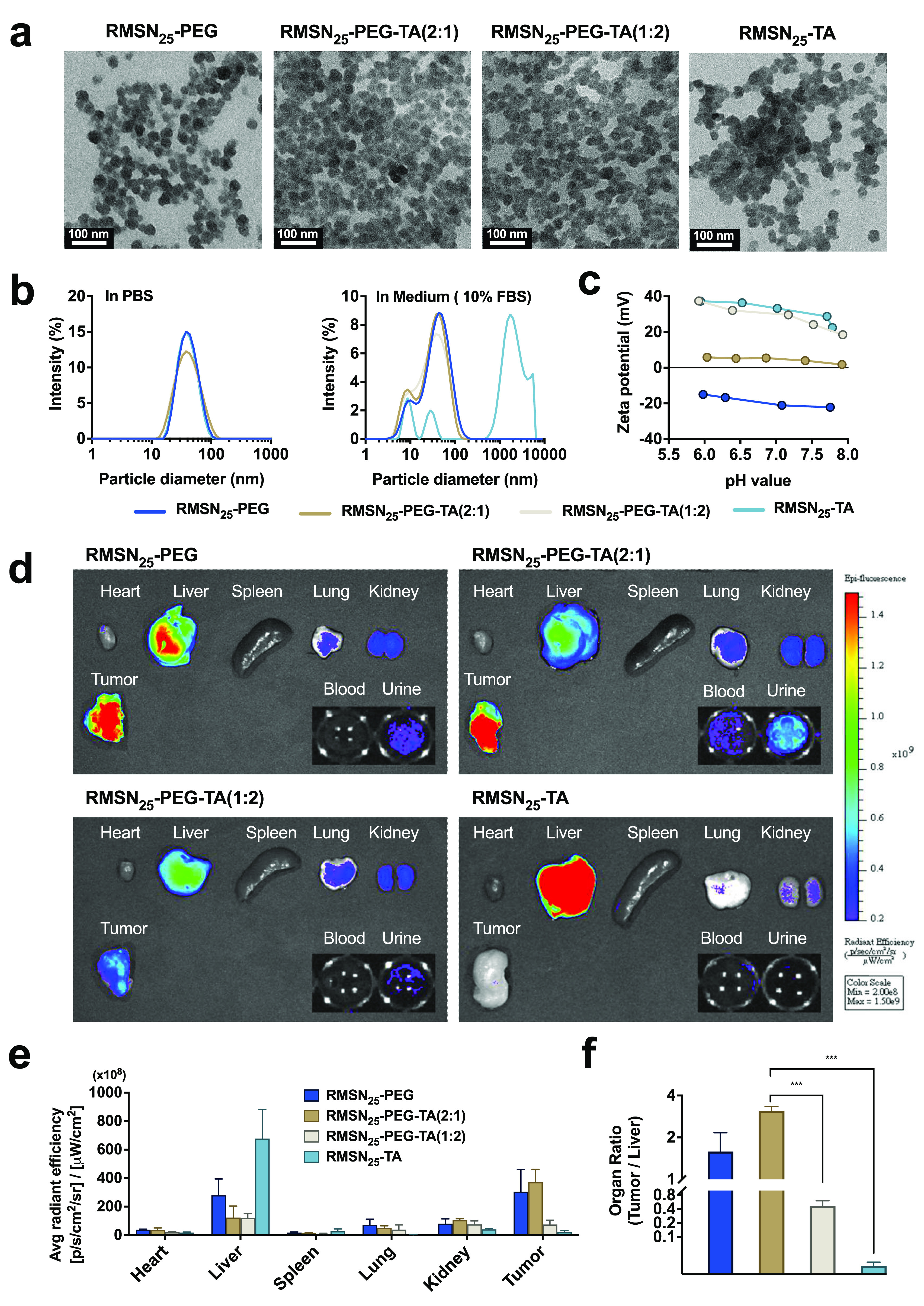
Characterization and biodistribution of various functionalized MSN_25_ in tumor-bearing mice. (a) TEM images of MSN_25_. (b) Hydrodynamic diameters of MSN_25_ in PBS and Dulbecco’s Modified Eagle Medium (DMEM) with 10% Fetal Bovine Serum (FBS). (c) Zeta potential values of MSN_25_ in PBS across a range of pH levels. (d) IVIS fluorescence images of organ tissues from 4T1 tumor-bearing mice, captured 24 h after injection with different RITC-labeled MSN_25_ types. (e) Quantitative analysis of fluorescence intensities. (f) Comparative assessment of the fluorescence signal ratio between tumor and liver tissues.

To evaluate effects of surface functionalization of 25 nm MSNs on the biodistribution and tumor-targeting ability, MSNs functionalized with various molar ratios of PEG groups to TA groups (PEG/TA) were synthesized. The obtained NPs were named RMSN_25_–PEG-TA(2:1) and RMSN_25_–PEG-TA(1:2), where the ratios of PEG/TA used were 2 and 0.5, respectively. As shown by TEM results (Figure 3a and Table S4), surface-modified MSNs had a uniform in morphology with respective mean particle sizes of 30.2 ± 3.6 nm, 29.4 ± 3.2 nm, 29.1 ± 3.2 nm, and 29.0 ± 4.4 nm for RMSN_25_–PEG, RMSN_25_–PEG-TA(2:1), RMSN_25_–PEG-TA(1:2), and RMSN_25_-TA, respectively. Also, the hydrodynamic diameters were consistently between 10 and 20 nm larger than the mean particle sizes as determined by TEM and the PDI, as shown in Figure 3b and Table S4, indicating no substantial aggregation of the NPs. However, RMSN_25_-TA revealed slight aggregation in TEM images and had a polydispersed size distribution in DLS measurements (PDI> 0.2). This can be attributed to the absence of PEGylation. In addition, DLS measurements in a serum-containing medium indicated deviations in hydrodynamic size due to the presence of serum proteins, with an additional peak at 10 nm and a shoulder peak at approximately 40 nm (Figure 3b). Notably, except for RMSN_25_-TA, which experienced particle aggregation, there was no significant increase in particle sizes in the presence of serum proteins compared to measurements in PBS, highlighting the colloidal stability of MSNs under corona protein conditions. The ζ-potentials of these samples were dependent on pH values (Figure 3c). At pH 7.4, ζ-potential values of RMSN_25_–PEG, RMSN_25_–PEG-TA(2:1), RMSN_25_–PEG-TA(1:2), and RMSN_25_-TA were −22, + 4, + 21, and +31 mV, respectively. The surface area and pore diameter decreased as the proportion of TA increased (Table S4), indicating that TA could functionalize the interior channels of the MSNs. Further confirmation was performed by an elemental analysis (Table S5). The obtained ratios of PEG/TA were 2.66 for RMSN_25_–PEG-TA(2:1) and 0.64 for RMSN_25_–PEG-TA(1:2), which were close to their respective theoretical values. A positive charge was associated with the nitrogen content of TA, which was roughly 3 times higher for RMSN_25_–PEG-TA(1:2) than that of RMSN_25_–PEG-TA(2:1) (1.08% vs 0.37%). We next investigated the biodistribution of the four types of MSNs in tumor-bearing mice (Figure 3d-f). In vivo imaging system (IVIS) imaging showed MSNs with PEGylation accumulate in tumor tissue, while those lacking PEG (RMSN_25_-TA) were mainly trapped in the liver. Intriguingly, RMSN_25_–PEG-TA(2:1) abundantly accumulated in tumors to give a tumor-to-liver ratio of 3.10 with an excellent EPR effect, whereas a stronger positive charge of RMSN_25_–PEG-TA(1:2) gave poorer tumor accumulation. Based on these results, we selected RMSN_25_–PEG-TA(2:1) for further drug carrying experiments.

### Overcoming the Limitations of BBB Penetration for Anticancer Drugs Using MSN_25_-PEG-TA Carriers in Brain Tumors

**Figure 4:**
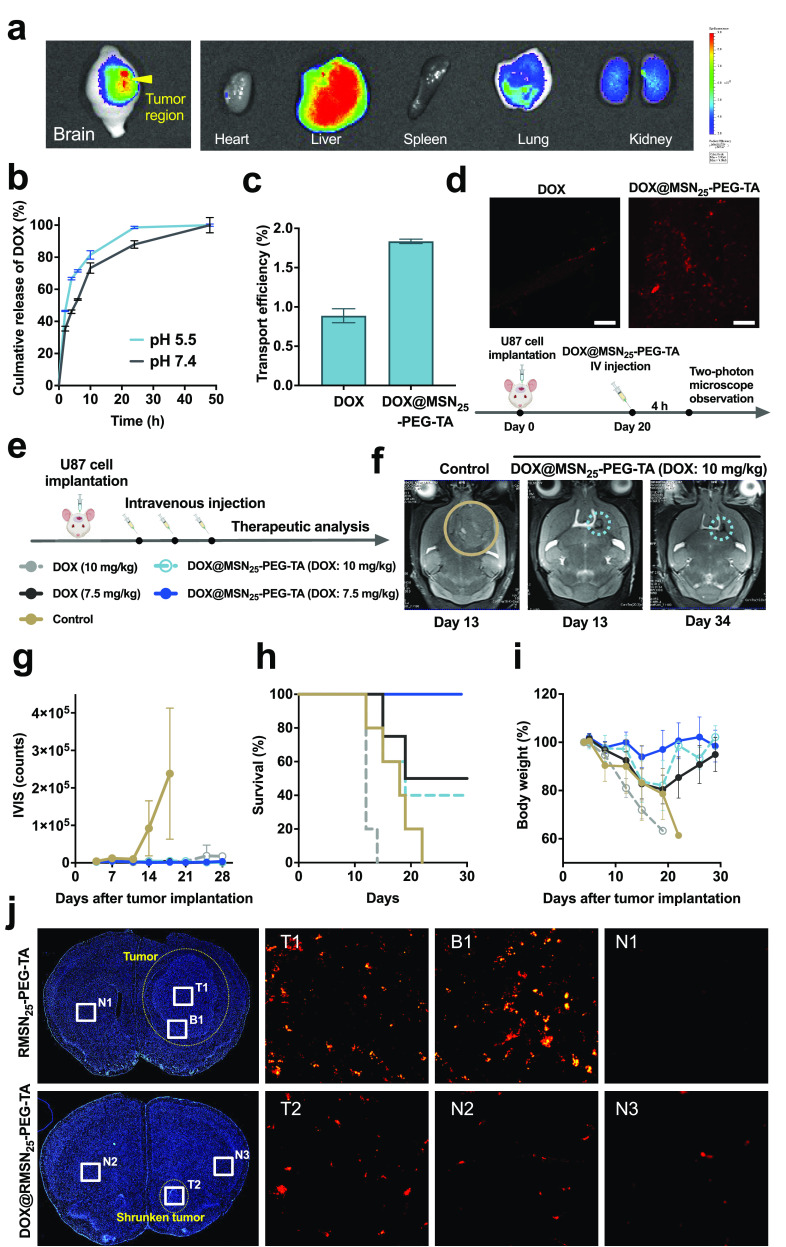
Biodistribution imaging, drug release, and therapeutic effects on mice orthotopically implanted with a U87 brain tumor. (a) Biodistribution images of RMSN_25_-PEG-TA obtained from an *in vivo* imaging system. (b) The *in vitro* doxorubicin (DOX) release of DOX@MSN_25_-PEG-TA at different pH values (pH = 7.4 and 5.5). (c) The transport efficiency of the *in vitro* blood–brain barrier (BBB) model incubated with DOX (10 μM) and DOX@MSN_25_-PEG-TA (an equivalent dose of 10 μM DOX) for 6 h was quantified by fluorescence spectrometry. (d) Diagrams of the experimental design (bottom panel). Representative multiphoton microscopy images of the brain tumor region of U87 orthotopic xenograft tumor-bearing mice (BALB/c nude) in the brain tumor region. After DOX or DOX@MSN_25_-PEG-TA administration with an equivalent dose of DOX (7.5 mg/kg body weight (BW)) for 4 h, red fluorescence indicated DOX localization. Scale bar = 70 μm. (e) Detailed experimental procedure of the therapeutic efficacy against mouse xenograft orthotopic gliomas (*n* = 5). Saline alone was used as the control group. (f) Magnetic resonance imaging (MRI) images of a control mouse brain and a brain with a U87 orthotopic glioma xenograft were administered with DOX@MSN_25_-PEG-TA at an equivalent dose of DOX (10 mg/kg BW). The circle indicates the position of the brain tumor. (g) Tumor size quantification based on luciferase intensity measured using an IVIS imaging system. (h) Overall survival. (i) BW variations. (j) Histological images of the brains of U87 orthotopic xenograft tumor-bearing mice treated with RMSN_25_-PEG-TA (200 mg/kg) or DOX@RMSN_25_-PEG-TA at an equivalent dose of DOX (10 mg/kg BW) following the same treatment procedure as in Figure 4e. The yellow arrowhead indicates the location of the tumor in the brain. Blue: nuclei (DAPI). Red: DOX/RITC. Upper left (RMSN_25_-PEG-TA treatment) and bottom left (DOX@RMSN_25_-PEG-TA treatment) responses before and after tumor shrinkage, respectively. Six different regions of interest were selected for further analysis, including the tumor areas (T1 and T2), the boundary between the tumor site and normal tissue (B1), and the normal brain areas (N1, N2, and N3).

We next explored the potential of using RMSN_25_–PEG-TA(2:1) as a carrier to deliver anticancer drugs into brain tumors to overcome the limitation of BBB penetration. To assess the in vivo drug distribution, we used orthotopic brain tumor models. Our findings, highlighted in Figure 4a, revealed a significant accumulation of RMSN_25_–PEG-TA(2:1) in both liver and brain tumor tissues. While a considerable concentration was observed in the liver, the key observation was the pronounced presence of the carrier within the brain tumor areas. Quantitative biodistribution in U87 brain tumor-bearing mice administered with RMSN_25_–PEG-TA(2:1) was determined through IVIS imaging (Figure S4a). To further support our findings, Figure S4b shows the effort to separate the tumor region from the entire brain, which includes both normal and tumorous areas. Despite the challenge in cleanly isolating the tumor due to the intricate nature of brain tissue, this distinct pattern of distribution underscores the potential of RMSN_25_–PEG-TA(2:1) for effectively targeting brain tumors, presenting a promising strategy to penetrate the BBB and deliver therapeutic agents directly to the tumor site. DOX is a highly effective anticancer agent and is used in a wide range of cancers. DOX can induce DNA damage by generating free radicals and intercalating into DNA, inhibiting topoisomerase II as a component of during DNA synthesis in cancer cells. Although DOX is a potent chemotherapeutic agent, its efficacy against brain tumors is hindered because its large, hydrophilic molecular structure and recognition by efflux transporters, such as P-glycoprotein, prevent it from effectively penetrating the BBB.

To measure the ability of MSNs to deliver DOX across the BBB, we synthesized DOX-loaded MSN_25_–PEG-TA (DOX@MSN_25_–PEG-TA) using the strong electrostatic interactions between MSN_25_–PEG-TA (without RITC conjugation) and DOX. Characteristics of DOX@MSN_25_–PEG-TA are shown in Table S6. After the DOX was loaded, the size of DOX@MSN_25_–PEG-TA by DLS measurements in phosphate buffered saline (PBS) was similar to that of MSN_25_–PEG-TA, indicating the drug-loading process did not affect the size or stability of the MSNs. The loading content (LC,%) and encapsulation efficiency (EE,%) for DOX@MSN_25_–PEG-TA were 3.55% and 71%, respectively.

Figure 4b shows the sustained release profile of DOX. The cumulative release of DOX from MSN_25_–PEG-TA under acidic conditions (pH 5.5) was faster than that in neutral conditions (pH 7.4) within 48 h. When the pH value was below the acid dissociation constant (p*K*_a_ = 8.22), the amine groups on DOX had a strong positively charged surface. These positively charged DOX molecules were attracted to the Si–O- of MSN_25_–PEG-TA (the pH value was adjusted with a sodium bicarbonate solution) through electrostatic forces. Under an acidic condition (pH 5.5), the surface charge on MSNs increased due to the Si–O- protonation and a higher amount of DOX was released from the MSN. This feature can support MSN_25_–PEG-TA achieving specific DOX release rates in the acidic tumor tissues. In addition, the DOX release rate is associated with the degradation of MSN, which undergoes hydrolysis and degradation in aqueous media. The degradation process and rate were examined by incubating MSN_25_–PEG-TA(2:1) and DOX@MSN_25_–PEG-TA(2:1) in PBS at 37 °C for 7 days. TEM and DLS analyses at various time points (Figure S5) revealed a gradual increase in degradation over time. The porous structure of MSN became unclear after 1 day of incubation, indicating degradation along the sidewalls of the pores. By day 3, particle aggregation was observed in TEM images and DLS results, suggesting defective PEGylation on the surface due to degradation. Almost all MSNs were degraded and exhibited severe aggregation by day 7. The count rate in light scattering, monitored by DLS, indicated a slow decline within the first day after incubation, followed by a rapid decline until the last measurement. These results indicated that MSN_25_–PEG-TA(2:1) and DOX@MSN_25_–PEG-TA(2:1) were biodegradable and mostly degraded within 7 days. The degradation of DOX@MSN_25_–PEG-TA(2:1) was associated with a slow drug release behavior. This characteristic supports the potential of MSN_25_–PEG-TA for specific DOX release in acidic tumor tissue. To further determine whether DOX@MSN_25_–PEG-TA could deliver DOX across the BBB, *in vitro* BBB model and *in vivo* mouse studies were again performed. Because all of the TEER values were larger than 100 Ω/cm^2^ after different treatments for 6 h (Figure S1b), the *in vitro* BBB integrity was considered to be similar to the *in vivo* BBB. Slightly less than 2% of DOX@MSN_25_–PEG-TA (10 μM) crossed the *in vitro* BBB model, which was greater than that when using 10 μM of DOX alone (0.88%), as shown in Figure 4c. Results indicate that MSN_25_–PEG-TA could favored the transportation of DOX with the potential to cross the BBB.

Figure S6 presents the results of a 24-h in vitro cytotoxicity assay, examining the impact of varying concentrations of DOX alone, MSN_25_–PEG-TA(2:1) nanoparticles, and DOX@MSN_25_–PEG-TA(2:1) nanoparticles on U87MG glioblastoma cells. The dose–response curve depicted in the graph provides a clear comparison of the cytotoxic effects, highlighting the therapeutic potential and safety profile of the nanoparticle formulations. To further explore the tumor-targeting, BBB penetration, and antitumor efficacies of DOX@MSN_25_–PEG-TA *in vivo*, luciferase-transfected U87 glioma cells were orthotopically implanted into nude mice to serve as a U87-LUC xenograft mouse model. As shown in Figure 4d, the red fluorescence was detected to show DOX localization in U87 xenograft tumor mice (housed for 20 days) by an intravenous injection of free DOX (7.5 mg/kg BW) and an equivalent DOX dose of DOX@MSN_25_–PEG-TA. At 4 h after being treated with DOX@MSN_25_–PEG-TA, numerous red DOX signals were observed in the brain tumor region by multiphoton microscopy when treated with DOX@MSN_25_–PEG-TA. On the contrary, no DOX signals were found when treated with free DOX, supporting our hypothesis that MSN_25_–PEG-TA contributed to BBB penetration and specific tumor accumulation via the EPR effect

Combining the advantages of an excellent EPR effect and BBB penetrating drug delivery of MSN_25_–PEG-TA, we determined its therapy efficiency against glioma tumors. Mouse xenograft orthotopic glioma (U87-LUC) were investigated with different treatments via tail vein intravenous administration every 4 days for a total of three times (Figure 4e), followed by magnetic resonance imaging (MRI) to monitor the brain tumor growth. T2-weighted MR images of mouse brains with different treatments are shown in Figures 4f, S7, and S8. The tumors were visible on the T2-weighted MR images of mouse brains in the control and MSN_25_–PEG-TA (750 mg/kg BW) groups. Tumor sizes with DOX@MSN_25_–PEG-TA treatment exhibited dramatic shrinkage (yellow arrowhead) on day 13 compared to treatment with the same dose of DOX (10 mg/kg BW). Also, the tumor region of DOX@MSN_25_–PEG-TA treatment was significantly smaller than that with free DOX alone. Furthermore, a comparison of the results of DOX@MSN_25_–PEG-TA from days 13 and day 34 revealed that the glioma tumor had been entirely suppressed. Finally, the tumors had almost disappeared by day 34. Moreover, tumor sizes were quantified based on the luciferase intensity using IVIS, resulting in a visible difference in tumor growth between the control and DOX@MSN_25_–PEG-TA groups in U87-LUC xenograft-bearing mice (Figure 4g).

To further assess the antitumor activity, 4 days after tumor implantation, the exact DOX dosages of free DOX and DOX@MSN_25_–PEG-TA were administered respectively through the tail vein three times at 4-day intervals. Overall survival (OS) and BWs were examined during the study period. As shown in Figure 4h, the survival of U87-LUC xenograft-bearing mice with DOX@MSN_25_–PEG-TA treatment at a dose of 7.5 mg DOX/kg BW was 100% at 28 days, compared to 50%, 40%, 0%, 0% for DOX (7.5 mg/kg), DOX@MSN_25_–PEG-TA (10 mg/kg BW), the control (no treatment), and DOX (10 mg/kg BW), respectively. In the experimental period, the high toxicity of DOX (7.5 and 10 mg/kg BW) weakened the mice and caused weight loss or even death. In contrast, the DOX@MSN_25_–PEG-TA-treated groups showed less or no toxicity and weight loss, implying that MSN_25_–PEG-TA could improve DOX-induced systemic toxicity and side effects (Figure 4i). Notably, the survival rate of mice treated with DOX@MSN_25_–PEG-TA at 7.5 mg/kg BW was superior to those treated with 10 mg/kg BW, suggesting that the higher dosage might surpass the maximum tolerated dose (MTD), where drug side effects and toxicity outweigh therapeutic efficacy.

Following the same treatment procedure in Figure 4e, histological micrographs of tissue sections of brain tumors are shown in Figures 4j and S9. Prior to tumor shrinkage, numerous red fluorescence signals of RITC from RMSN_25_–PEG-TA (200 mg/kg BW) were readily observed in the tumor region (T1) and near the tumor boundary (B1). In contrast, no signals were detected in the normal region of the brain (N1). We proposed that most of the RMSN_25_–PEG-TA had initially accumulated inside the brain tumor due to a strong EPR effect, leading to no observation of MSNs in the normal brain before tumor shrinkage. U87-LUC xenograft-bearing mice were treated with DOX@RMSN_25_–PEG-TA (DOX: 10 mg/kg BW) to suppress the tumors. After the tumor had shrunk, red signals of the DOX@RMSN_25_–PEG-TA were distributed in the entire brain region, including both the tumor and normal brain, and had remarkably declined but were still clearly visible (T2, N2, and N3). Results suggested that DOX@RMSN_25_–PEG-TA had crossed the BBTB during the later stages of brain tumor treatment. Taken together, the therapeutic effect of DOX@RMSN_25_–PEG-TA was attributed to excellent tumor-targeting capability via the EPR effect, accompanied by DOX release and BBTB penetration.

**Figure 5:**
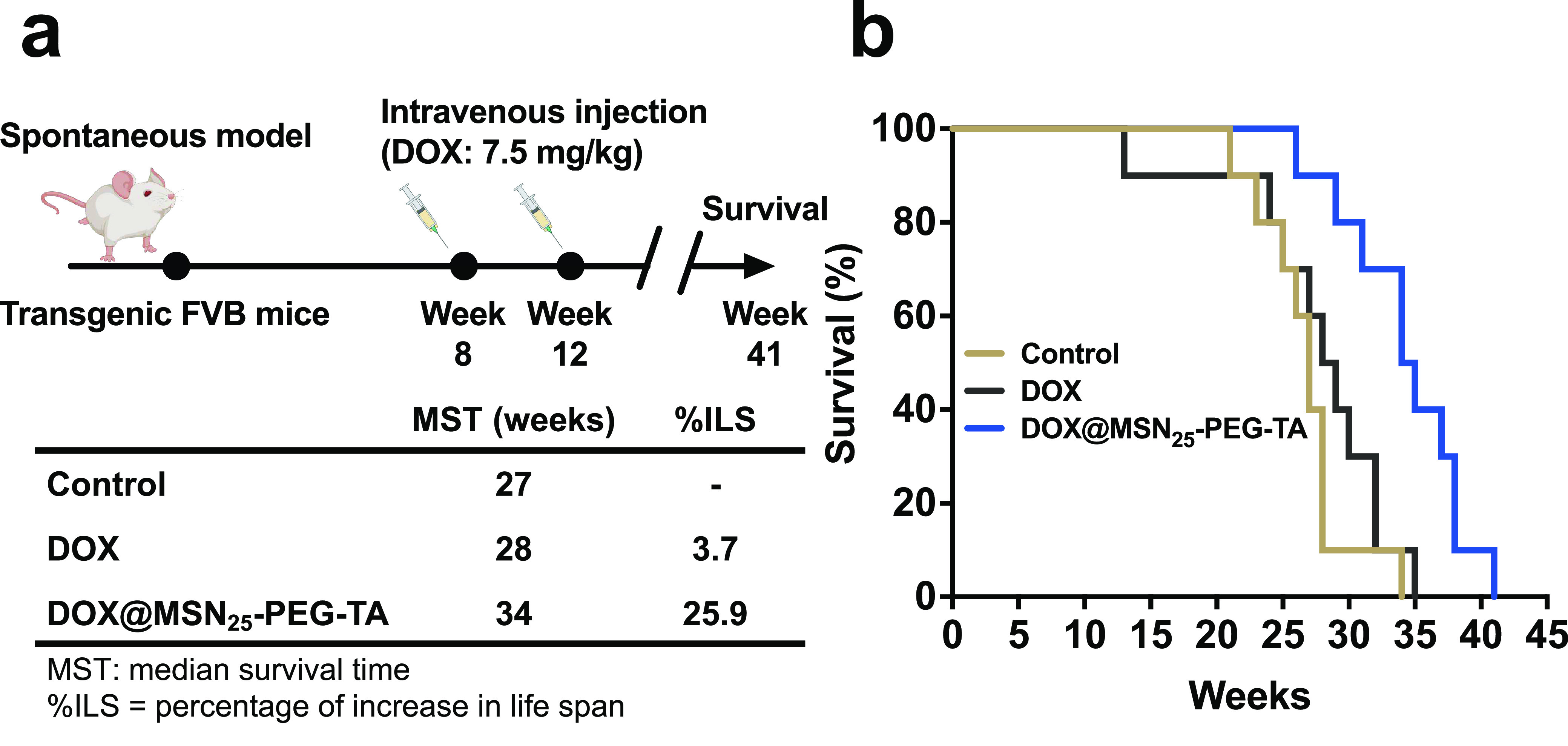
Therapeutic effects on transgenic FVB mice with a spontaneous brain tumor. (a) Schematic diagram of the experimental design in transgenic FVB mice with spontaneous brain tumors that received an intravenous injection of doxorubicin (DOX; 7.5 mg/kg body weight) and an equivalent DOX dose of DOX@MSN_25_-PEG-TA for three times at 4 day intervals in weeks 8 and 12. Saline alone was used as the control group. Kaplan–Meier plots of overall survival. (b) Median survival time (MST) and percent increase in life span (% ILS) of mice with spontaneous brain tumors.

In addition to the orthotopic tumor model, we further studied spontaneous brain tumors in mice, which is a better model system that mimics the natural integrity of the BBB.^46^ To evaluate the therapeutic efficacy of DOX@MSN_25_–PEG-TA, transgenic FVB mice with engineered spontaneous brain tumors were treated with DOX@MSN_25_–PEG-TA or DOX alone at the same dose of DOX (7.5 mg/kg BW) by intravenous injection for three times at 4-days intervals at weeks 8 and 12 (Figure 5a). With DOX@MSN_25_–PEG-TA treatment, the OS of mice significantly increased as compared to the control and DOX-alone groups. In addition, the survival analyses were performed by measuring the median survival time (MST) and percent increase in life span (% ILS), which are standard criteria when conducting the preclinical survival studies (Figure 5b). The MST of control mice was 27 weeks. Administration of free DOX (7.5 mg/kg BW) did not effectively increase mice survival (MST = 28 weeks,% ILS = 3.7%), whereas DOX@MSN_25_–PEG-TA with an equivalent dose of DOX significantly prolonged the animal MST to 34 weeks (% ILS = 25.9%). In summary, these results in the spontaneous brain tumor model provide strong evidence of the therapeutic potential of using DOX@MSN_25_–PEG-TA to improve therapeutic outcomes for brain tumors over DOX alone.

### Preclinical Safety: Single-Dose Study

**Figure 6:**
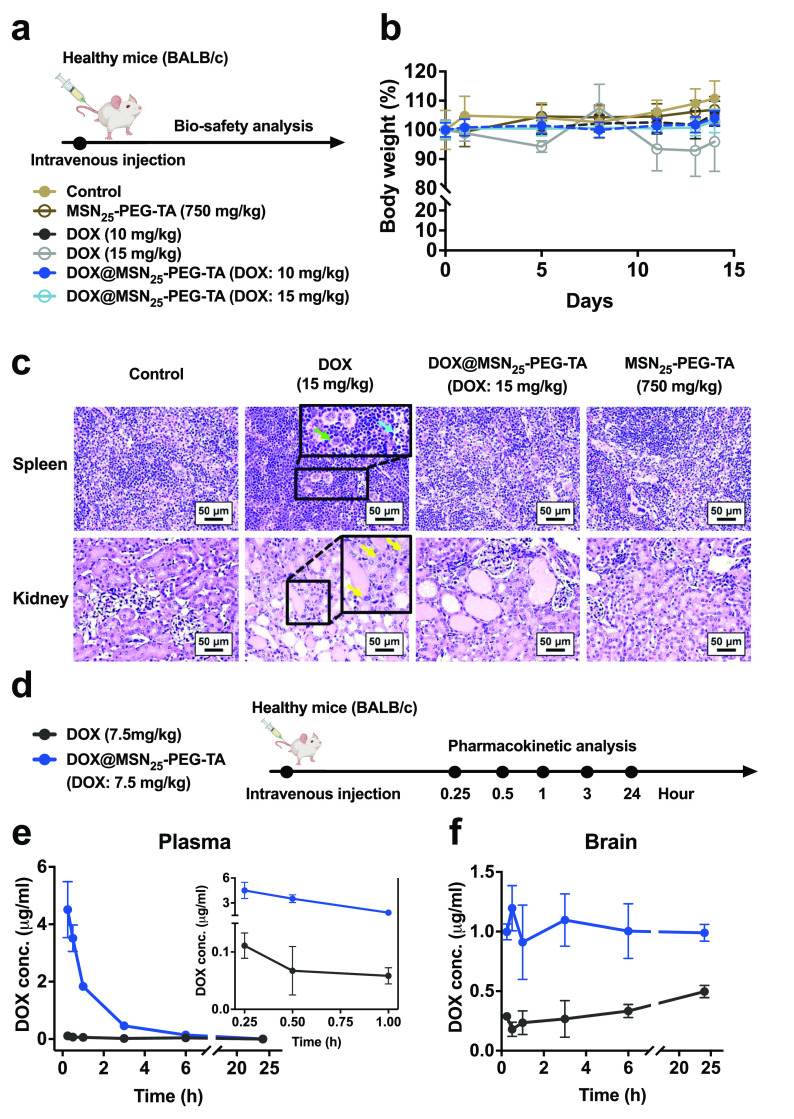
Toxicity and pharmacokinetic studies of DOX@MSN_25_-PEG-TA in healthy mice. (a) Schedule of the single-dose toxicity study. Healthy BALB/c mice were injected intravenously with different doses of DOX alone (10 and 15 mg/kg BW), DOX@MSN_25_-PEG-TA (equivalent DOX dose), and MSN_25_-PEG-TA (750 mg/kg BW), respectively. (b) BW variations in mice with different treatments. (c) Representative histopathological analysis of the spleen and kidneys in healthy BALB/c mice. Hematoxylin and eosin (H&E)-stained images of mice after treatment with DOX alone (15 mg/kg BW), DOX@MSN_25_-PEG-TA (DOX: 15 mg/kg BW), or MSN_25_-PEG-TA (750 mg/kg BW). Splenic extramedullary hemopoiesis (green arrowhead), lymphocytic apoptosis (cyan arrowhead), and renal hyaline cast and tubular regeneration (yellow arrowhead) are presented, respectively. Scale bar = 50 μm. (d) Schedule of pharmacokinetic study. Healthy BALB/c mice were intravenously injected with a single dose of DOX alone (7.5 mg/kg BW) or DOX@MSN_25_-PEG-TA (DOX: 7.5 mg/kg BW) for one time. Concentration–time curves of DOX in the plasma (e) and brain (f) of mice were determined by fluorescence spectrophotometry at the indicated times.

Systemic toxicity and the maximum dosage of the DOX@MSN are critical issues for clinical translation. To address these issues, we focused on a non-Good Laboratory Practice (non-GLP) single-dose toxicity study conducted in healthy BALB/c mice with a 14-day schedule (Figure 6a). Mice were injected intravenously with different doses of DOX alone (10 and 15 mg/kg BW), DOX@MSN_25_–PEG-TA (equal to DOX: 10 and 15 mg/kg BW), and MSN_25_–PEG-TA (750 mg/kg BW), respectively, to evaluate the biosafety and biocompatibility by recording the BW changes, blood assays, and toxicological histopathological analyses during the experimental period. Figure 6b shows that mice injected with DOX alone (15 mg/kg BW) exhibited severe weight loss (of >15%) and other signs of unacceptable toxicities, such as ascites. However, there was no weight loss in the group treated with DOX alone at 10 mg/kg BW, indicating limitations of free DOX dose escalation. At the same time, the maximum tolerated dose of DOX was about 10–15 mg/kg BW.

The most important finding was the BW of the mice in the DOX@MSN_25_–PEG-TA (DOX: 15 mg/kg BW) group showed no abnormalities during the study period, implying that the drug-loaded NPs were able to improve the drug-induced toxicity. A high dose of MSN_25_–PEG-TA (750 mg/kg BW) did not affect BW changes, implying that DOX carried in MSN_25_–PEG-TA caused less systemic toxicity while suppressing tumor growth.

We further confirmed this lesser systemic toxicity in DOX@MSN_25_–PEG-TA by examining the complete blood count (CBC) and blood biochemical analyses, as shown in Tables S7 and S9. Mouse blood was collected at the end point of the experimental design. DOX treatment-related toxicities were dose-dependent for both free DOX and DOX@MSN_25_–PEG-TA at the two concentrations, whereas all the indices had recovered to normal levels when treated with DOX@MSN_25_–PEG-TA (DOX: 10 mg/kg BW) compared to DOX alone (10 mg/kg BW). Even when the maximum tolerated dose of DOX (15 mg/kg BW) was administered, some alterations in CBC and blood biochemical analyses were still achieved by using DOX@MSN_25_–PEG-TA (DOX: 15 mg/kg BW) (Table S7 and S10). It appeared that DOX@MSN_25_–PEG-TA was effective in improving systemic toxicity caused by DOX. As a first-line clinical anticancer drug, one of the major concerns with DOX is its toxicity-induced side effects. To further verify whether MSN_25_–PEG-TA caused a reduction in DOX toxicity, toxicological histopathological analysis of major organs, such as the heart, liver, spleen, lungs, kidneys, and brain, were thus processed with fixation, tissue sectioning, and hematoxylin and eosin (H&E) staining (Figures 6c, S10). We noticed that 15 mg/kg BW of DOX caused lesions with visible pathological changes in the spleen and kidneys, as indicated by the splenic extramedullary hemopoiesis (green arrowhead in the enlarged graph) and lymphocytic apoptosis (cyan arrowhead in the enlarged graph) and pronounced renal hyaline cast and tubular regeneration (yellow arrowhead in the enlarged graph). The severity was also graded as moderate to moderately high. In contrast, with an equivalent DOX dose (15 mg/kg BW) of DOX@MSN_25_–PEG-TA treatment, there was no apparent toxicity, and all of the organs had typical histological structures. The severity of damage to the spleen and kidneys was graded as minimal to mild. Therefore, these results revealed that DOX@MSN_25_–PEG-TA is significantly favorably improved the severe lesions seen after treatment with DOX alone.

Based on the results of the toxicity-related studies, a safe dose of DOX@MSN_25_–PEG-TA (DOX: 7.5 mg/kg BW) was used for the following PK study assayed by quantifying the DOX concentrations. Healthy mice were intravenously injected once with DOX alone or DOX@MSN_25_–PEG-TA at the same DOX dose (7.5 mg/kg BW). DOX concentrations of in the brain and plasma at different time points (0.25, 0.5, 1, 3, and 24 h) post intravenous administration were investigated using fluorescence spectrometry (Figure 6d). As shown in Figure 6e, DOX@MSN_25_–PEG-TA exhibited significant enhancement of blood retention within 3 h in mice due to its long circulation time. However, DOX in mice treated with free DOX was rapidly cleared out of the bloodstream, and it had returned to an undetectable level after 0.25 h (Figure 6e, inset curve). Notably, at 24 h after the injection, DOX@MSN_25_–PEG-TA maintained a higher DOX concentration inside the brain, and the concentration of DOX corresponding to DOX@MSN_25_–PEG-TA was nearly 6.6-times higher than that of free DOX at 0.5 h. In contrast, DOX treatment did not produce a noticeable increase in DOX in brain tissues, which was close to 0.2 to 0.4 μg/mL (Figure 6f). This evidence indicates that the enhanced accumulation of DOX in the brain when delivered with DOX@MSN_25_–PEG-TA could be attributed to its prolonged circulation time and the decreased clearance associated with MSN_25_–PEG-TA.

### Comprehensive Analysis of Protein Corona Composition and Its Impact on MSN–BBB Interaction *in Vivo*

**Figure 7:**
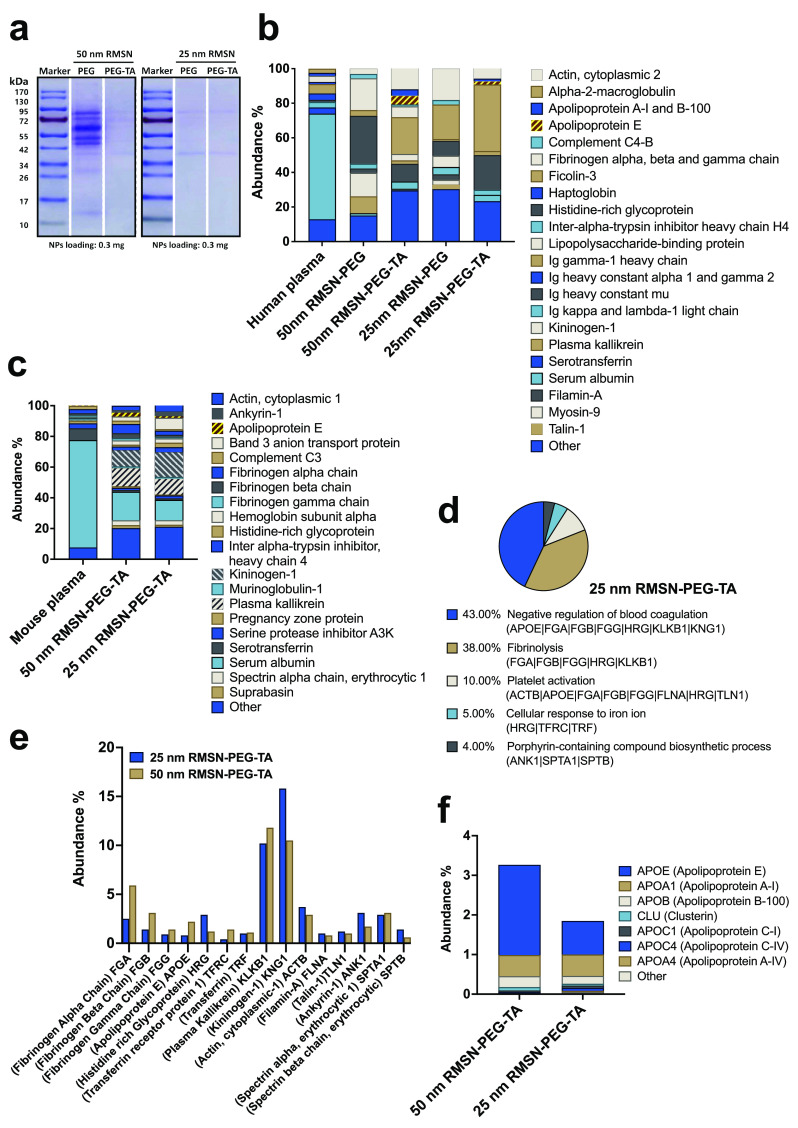
Protein corona analyses of RMSNs obtained from (a, b) *in vitro* human plasma and (c–f) *in vivo* mouse plasma. (a) One-dimensional SDS-PAGE gel visualization of proteins adsorbed onto 50 and 25 nm RMSN-PEG and RMSN-PEG-TA after a 30 min incubation with human plasma. (b) Predominant corona proteins adsorbed on various RMSNs in *in vitro* human plasma, as characterized by LC-MS/MS. (c) Predominant corona proteins identified for 50 and 25 nm RMSN-PEG-TA identified using LC-MS/MS in an *in vivo* mouse model. (d, e) Gene ontology (GO) enrichment of biological functions based on proteomic analyses of proteins associated with the RMSN-PEG-TA corona. This includes (d) a pie chart representation and (e) protein abundances in the GO enrichment pathway. (f) Lipoprotein composition of the protein corona of RMSN-PEG-TA.

Next, we looked for the possible reasons behind the advantageous profile of our MSN_25_–PEG-TA. Since our fabricated MSNs carried no targeting ligand, we suspect that the protein corona which had strongly adsorbed onto circulating MSNs helped them cross the BBB.^47^ To assess this, we conducted a detailed analysis examining the impact of incorporating TA and varying the nanoparticle size, comparing 25 to 50 nm. This hypothesis was initially validated through *in vitro* characterizations using human plasma and later substantiated *in vivo* using murine models. First, a thermogravimetric analysis (TGA) quantified the weight percentages of the corona proteins in human plasma associated with various NPs in *in vitro* studies. Specifically, for RMSN_50_-PEG, it was 4.13 ± 0.25 wt % and for RMSN_50_-PEG-TA, it was 0.88 ± 0.24 wt %. Corona proteins of 25 nm MSNs, both RMSN_25_–PEG and RMSN_25_–PEG-TA, were not detectable by the TGA. These TGA data emphasize the pivotal roles of NP size and surface modifications in influencing protein adsorption onto RMSNs. Notably, by introducing TA and simultaneously reducing the particle size, RMSN_25_–PEG-TA proved to be the NPs with minimal protein adsorption, consistent with sodium dodecyl sulfate polyacrylamide gel electrophoresis (SDS-PAGE) findings (Figure 7a). Drawing from a recent study, it was noted that when NPs absorb copious amounts of blood proteins, only about 27% retain their target-binding efficiency.^48^ This reduction stems from the corona architecture, which is not a monolayer but rather an assembly of interlinked proteins. Subsequent quantitative liquid chromatographic tandem mass spectroscopic (LC-MS/MS) analyses of the 50 nm MSN-PEG, with a relative abundance of 27.7% of histidine-rich glycoprotein and 18.4% of fibrinogen protein, gave the largest amount of protein corona among the tested NPs (Figure 7b).

To identify the critical biological impacts of RMSN-PEG-TA on *in vivo* protein corona formation, we conducted an exhaustive comparison using mouse models. Here, we hypothesized that some of the monolayered targeting proteins on RMSN-PEG-TA could bind to brain endothelial receptors and initiate the transport of MSNs across the BBB, either through the paracellular route or by transcellular internalization. According to quantitative proteomic analyses, the *in vivo* corona protein composition significantly differed from plasma proteins. Additionally, there were observable similarities between the 25 and 50 nm RMSN-PEG-TA (Figure 7c). Unsurprisingly, serum albumin, known for extending the circulation duration, was identified as one of the predominant proteins. Given that many tumors overexpress albumin-binding proteins such as SPARC and gp60, researchers have investigated the potential of albumin NPs for biomimetic delivery targeting brain tumors.^49,50^

## Discussion

### Concept of the Blood–Brain Barrier (BBB)

The BBB is a vital physiological barrier in the CNS that regulates the movement of ions and molecules from circulating blood into the brain. It protects the brain from invading pathogens and toxic agents. The BBB also prevents drug molecules from entering the brain to treat specific brain diseases. More than 95% of drugs cannot achieve a therapeutic dose in the brain. NPs conjugated with targeted ligands that bind to receptors on endothelial cells, such as human H-ferritin, ApoE, and lactoferrin, may promote BBB penetration.^51−53^ However, modifications with targeted ligands on the exterior surface may also affect the suspension and circulation of NPs in the blood and accelerate the clearance of NPs. Hence, researchers must pay attention to developing simple but therapeutic NPs, which may have better clinical translation potential.

### Enhanced Permeability and Retention (EPR) Effect

The microvasculature around a tumor differs from their normal counterparts, which typically contains tightly joined endothelial cells and prevents the entrance of molecules from outside the blood vessels. In most growing solid tumors, hyperactive angiogenesis leads to a leaky vasculature and reduced lymphatic drainage, facilitating the passive accumulation of NPs without additional modifications. This phenomenon is referred to as the EPR effect.”^54^ Hence, NPs targeting tumors via the EPR effect provide an excellent opportunity to tackle poor tumor selectivity. EPR-mediated passive tumor targeting relies on the physicochemical properties of NPs, including the particle size, surface modifications, charge, chemical compositions, etc.^54^ In addition, the ability to cross the BBB is depends on several physicochemical properties of the NPs in physiological conditions: (1) the effect of particle size on the BBB transport efficiency; (2) the effect of the surface charge on BBB penetration; and (3) upon exposure to biological fluids, serum proteins adsorbed onto the surface of NPs, forming a physicochemical identity which is known as the “protein corona” effect.^55^ This protein layer surrounding the NPs consequently influences their fate and therapeutic/diagnostic performance.

### Extending the Circulation Time of NPs

Another critical issue in nanomedicine is the reorganization of the reticuloendothelial systems (RES), such as phagocytic cells and Kupffer’s cells, which can perform the eliminate of foreign NPs. A shielding strategy by introducing PEG, a hydrophilic polymer, is the most commonly used approach to increase the long half-life of NPs in circulation. Through the covalent conjugation of PEG chains to NPs, PEGylation can effectively reduce renal clearance and immunogenicity due to steric hindrance; it may prevent the rapid depletion of NPs from the bloodstream, resulting in longer circulatory times when administered intravenously. Typically, the use of high-molecular-weight PEG (Mw > 2000) is known to cause an immunological response of anti-PEG antibodies that restrict the therapeutic effect of PEGylated NPs and are accessible to rapid elimination.^56^ Moreover, dense coatings with low–molecular-weight PEG might allow larger NPs to penetrate the brain parenchyma.^57^ In this study, we explored the size and charge effects of PEGylated MSNs on BBB delivery and brain tumor targeting.

### BBB Penetration of Silica NPs

Previous evidence demonstrated that PEGylated silica NPs (PSiNPs) with a diameter of 25 nm showed higher uptake efficiency in the brain capillary endothelial cells than did 100- and 50 nm PSiNPs.^58^ These results indicate the potential application of small-sized silica NPs for delivering diagnostic and therapeutic agents across the BBB.^58^ Another study suggested that surface charge is essential in allowing NPs to across the BBB. Cationic and high concentrations of anionic nanoparticles could change the BBB integrity and permeability because of charge-induced toxic effects. However, neutral and low concentrations of anionic NPs were not distributed into the BBB, decreasing their potential for utilization as a drug carrier for brain therapy.^59,60^ Previous research also evaluated that the protein corona is associated with NP transportation across the BBB, emphasizing that the protein surrounding the NPs can significantly alter the therapeutic or diagnostic performance.^55^ An ideal NP suitable for BBB penetration should rely on the following parameters: a smaller size, near-neutral charge, decreasing protein corona adsorption, and long circulation time.

### Critical Features of MSNs for Tumor Therapy

According to a review paper that surveyed all published data between 2006 and 2016, less than 1% of NPs injected into the animals could reach solid tumors.^61^ The main reasons for the low delivery efficiency were the mononuclear phagocytic system and renal clearance, which removed 99% of the administered NPs. Such low delivery efficiency hinders the applications of NPs for cancer therapy. Hence, there is an urgent need to develop strategies for better tumor-targeting by NPs. Generally, the organ NP distribution ratio of tumor to liver (tumor/liver) based on the IVIS signals is an important criterion when judging the quality of the EPR effect. We have demonstrated that injected RMSN_25_–PEG-TA(2:1) could reach the solid tumors within 24 h after a tail vein injection with a tumor/liver organ ratio exceeding 3 (Figure 3f), the delivery efficiency of which was about 6 times (values of organ ratios reported in the literature were about 0.3 to 0.7) better than literature data we surveyed.^62−65^ Results demonstrated that specific ranges of the surface charge and PEG/TA-silane ratio might be critical parameters for enhancing the tumor-targeting ability of NPs. Also, we noted the higher tumor accumulation of MSN_25_–PEG-TA(2:1) could have been due to its longer circulation time in the blood.

In summary, materials design strategies based on engineered sizes, charges, and surface properties of NPs could overcome these challenges, including (1) increasing the circulation half-life more efficiently in the bloodstream by shielding with short PEG (Mw 500), (2) promoting specific passive accumulation within the tumor via the EPR effect, and (3) crossing to the BBB. We developed small-sized MSNs (25–30 nm) with specific surface properties capable of penetrating the BBB and delivering bioactive payloads into the brain for brain tumor therapy.

### Therapeutic Bottleneck of Brain Tumor

Brain cancers can originate in the brain (primary brain tumors) or from another part of the body and metastasize to the brain (metastatic tumors). Prognoses of primary and metastatic brain cancer patients are generally poor. The median survival duration is less than a year after beginning treatment, including surgery, radiation, chemotherapy, and their combinations. For instance, glioblastomas, one form of primary brain cancer, can recur within the peritumoral region, even when the BBB remains intact. This significantly limits the efficacy of chemotherapeutics to eradicate the remaining infiltrating cancer cells. As shown in Figures 4j and S9, the EPR and BBB penetration capability of NPs were evaluated. When the tumor became smaller, DOX@MSN_25_–PEG-TA not only accumulated in the tumor area but also appeared in the normal brain regions, meaning that the EPR effect was reduced and did not contribute much to tumor targeting. Many RMSN_25_–PEG-TA, therefore, oenetrated to BBB. DOX@MSN_25_–PEG-TA crossing the BBB wass easier to observe. Thus, we proposed that in addition to the excellent EPR effect, DOX@MSN_25_–PEG-TA can cross the BBB during the late stage of brain tumor treatment, thereby offering improved therapeutic efficiency against brain tumors with an intact BBB. The combination of dual functions could greatly benefit brain tumor therapy.

### Doxorubicin (DOX) in Cancer Treatment

To date, only a few drugs have been approved for treating brain cancers. To a significant extent, this has more to do with the BBB blocking the entry of most chemotherapeutic agents instead of brain tumor cells being inherently resistant to them. DOX is a well-known and widely used anticancer drug approved for treating various cancers but not for brain cancers.^66,67^ However, DOX has shown therapeutic efficacy against malignant brain cancer cells *in vitro*. Moreover, DOX appears to be a safe and very effective treatment of malignant brain tumors when injected intratumorally in clinical trials but not intravenously.^68^ DOX cannot distinguish between cancerous cells and normal cells. Its myocardial toxicity, multiple drug resistance (MDR), and narrow therapeutic index result in serious side effects, limiting its clinical application.^69^ It is crucial, therefore, to improve the side effects of DOX, and developing brain tumor therapeutic approaches has become an urgent clinical need. Notably, the better therapeutic activity and fewer adverse effects of DOX@MSN_25_–PEG-TA led to prolonged OS, compared to DOX alone (Figure 4h).

### Biosafety of Mesoporous Silica Nanoparticles (MSNs)

MSNs are composed of amorphous silica, which is known to be biocompatible and biodegradable. They are excreted through feces or urine in a partially degraded form or dissolved silica species (silicic acid). The US Food and Drug Administration (FDA) considers silica a GRAS substance (Generally Recognized as Safe). More recently, the ultrasmall silica NP “C’Dot Drug Conjugates” to enter phas 1/2 clinical trials for patients with solid tumors that overexpress folate receptor alpha (FRα).^70^ So far, there has been no report of any safety issues. PEGylated MSNs are generally considered nontoxic due to their excellent biocompatibility.^71^ Hence, in this study, we developed MSNs as drug delivery carriers offering significantly enhanced therapeutic efficiency in mouse models of GBM. A non-GLP single-dose toxicity study of a 14-day was schedule conducted to investigate the biocompatibility and biosafety of MSN and DOX-loaded MSNs. Results suggested biocompatibility, no toxicity, and no treatment-related histopathological changes were observed in mice administered with MSN_25_–PEG-TA (Figures 6a-c, S8, Tables S7, S9). DOX@MSN_25_–PEG-TA are not only biocompatible but can reduce the DOX toxicities, resulting in a broader therapeutic window than original DOX drugs. MSN_25_–PEG-TA successfully improved DOX-induced cytotoxicity, which can be beneficial in clinical use. In addition, in combined with the EPR effect, DOX@MSN_25_–PEG-TA caused a highly favorable distribution of DOX delivered to brain tumor sites while avoiding toxicities toward normal tissues.

### Insights into Protein Corona and Potential Pathways for BBB Penetration

Building upon our findings from the LC-MS/MS analyses which revealed a substantial protein corona on the 50 nm MSN-PEG, we observed that this significant presence may not have led to optimal targeting protein binding due to its interconnected nature. However, RMSN-PEG-TA offers a distinct difference with less protein adsorbed and a possible near monolayer, a structural arrangement that likely influences MSN-cell interactions more directly. Our analyses further revealed that RMSN-PEG-TA had a preference for proteins with a molecular weight between 20 and 60 kDa (Figure S11a) and predominantly bound to proteins with a pI of 5–6, as showcased in Figure S11b. Notably, apolipoprotein E (ApoE) was consistently detected in both the 50 and 25 nm RMSN-PEG-TA (Figure 7b).

Beyond serum albumin, high relative abundances of plasma kallikrein and kininogen were observed. Interestingly, plasma kallikrein and kininogen are components of the kallikrein-kinin system (KKS), which influences both inflammatory processes and beneficial mechanisms like vasodilation and tissue repair.^72^ When activated, plasma kallikrein cleaves high-molecular-weight kininogen (HMWK or kininogen-1) to release bradykinin (BK), a potent mediator that can increase the permeability of the BBB, making it more “leakier.″^73,74^ While plasma kallikrein and kininogen-1 are not directly involved in the immediate function of the BBB by affecting tight junctions, their influence on its permeability can notably impact brain therapeutic strategies. A gene ontology (GO) analysis revealed that 43% of the adsorbed proteins, including ApoE, plasma kallikrein, and kininogen, are involved in the negative regulation of blood coagulation, highlighting a potential anticoagulant influence of these bound proteins (Figures 7d, 7e). Furthermore, ApoE, identified in our *in vitro* human plasma studies, was also evident in the *in vivo* evaluation. (Figure 7f).^75,76^ Notably, ApoE plays a crucial role in brain cholesterol transport, ensuring proper neural function by its high affinity for the low-density lipoprotein receptor (LDLR) and several low-density lipoprotein-associated receptors (LRPs). As LRPs are highly expressed by brain endothelial cells, ApoE has become one of the leading candidates as a targeting ligand of NPs for drug delivery to the brain.^33^

To sum up, we propose that the minimum monolayer-like protein corona of RMSN_25_–PEG-TA allowed its adsorbed proteins to be “visible” to the cell receptors. Our detailed proteomic analyses of the protein corona highlight multiple pathways for BBB penetration by RMSN_25_–PEG-TA. Specifically: (1) ApoE interactions facilitate receptor-mediated transcytosis via the transcellular route; (2) serum albumin underscored adsorptive-mediated transcytosis, also through the transcellular pathway; and (3) the kallikrein-kinin system potentially altered tight junction permeability, linked to the paracellular route. Taken together, these insights suggest that RMSN_25_–PEG-TA provides a simple but versatile design approach for targeted brain delivery of NPs.

## Conclusions

The drug delivery efficiency and biological barriers (intra/extracellular barrier and serum protein corona effect) are still critical issues that restrict the therapeutic development of cancer nanomedicines. The present work demonstrated a simple strategy using MSNs to target brain tumors via the EPR effect and BBB penetration by designing the diameter and the ratio of PEG molecules (short PEG with Mw 500) and surface charged molecules (positively charged TA-silane). Results suggested that the small size (25 nm), near neutral charge (+4 mV), and a specific ratio of PEG to TA-silan groups (2:1) favored MSNs crossing the BBB both *in vitro* and *in vivo*, and also promoted the ability of to target tumors based on the EPR effect. After DOX loading, the therapeutic MSNs significantly enhanced the pharmacokinetics changes of DOX in the plasma and the brain, enabling the delivery of DOX to the brain through BBB penetration, accompanied by the suppression of brain tumor growth with the improvement of DOX-induced severe side effects. In both xenografts and spontaneous mouse models of brain tumors, DOX@MSN treatments showed prolonged survival rates, indicating that DOX-loaded MSNs may improve therapeutic outcomes with the potential of being a clinical brain tumor drug.

Furthermore, it is of paramount importance to investigate the role and underlying molecular mechanisms of corona proteins in regulating BBB/BBTB permeability. Carefully designed drug@nanocarriers, capitalizing on the protein corona to help BBB permeability, offer a promising approach for treating brain diseases.

## Experimental Section

### Materials

All chemicals were used without additional purification. Fluorescein isothiocyanate (FITC)-dextran, molecular weight (Mw) 70 kDa, rhodamine B isothiocyanate (RITC), and 2-mercaptoethanol were purchased from Sigma-Aldrich. Cetyltrimethylammonium bromide (CTAB, 99+%), tetraethyl orthosilicate (TEOS, 98%), and ammonium hydroxide (NH_4_OH, 28–30 wt %) were purchased from Acros. 2-[Methoxy(polyethyleneoxy)6–9propyl]trimethoxysilane (PEG-silane, Mw 459–591 g/mol), trimethoxysilylpropyl-N,N,N-trimethylammonium chloride (TA-silane, 50% in methanol) and (3-trihydroxysilyl)propylmethylphosphonate (THPMP-silane, 42% in water) were acquired from Gelest. Dulbecco’s Modified Eagle Medium (DMEM) was purchased from Gibco Co. Fetal bovine serum (FBS) were purchased from HyClone, GE. An anti-CD31 antibody (BS1574) was purchased from Bioworld, an anti-CD140b antibody (16–1402–82) was purchased from Invitrogen, and an anti-ZO-1 antibody (ab221547) was purchased from Abcam. Secondary goat antirabbit IgG-FAM 488 (TAFB02-F) was purchased from BioTnA. Ethanol at 99.5% was purchased from Choneye Pure Chemicals. Doxorubicin (DOX) was obtained from Scinopharm Taiwan Ltd.

### Synthesis of Positively and Negatively Charged PEGylated MSNs

PEGylated MSNs (MSN-PEG) incorporating with a fluorescent dye (RITC) were synthesized by a method described in a previous studies.^77−79^ In short, 0.29 g of surfactant (CTAB) was dissolved in 150 mL of an ammonium hydroxide solution at 60 °C in a sealed beaker. Then, under vigorous stirring, 0.88 M ethanolic TEOS and fluorescent dye (RITC-APTMS) were added to the solution. For positively charged surface modification, 1.09 mmol of PEG-silane with 0.54 and 2.2 mmol of TA-silane (with ratios of PEG/TA of 2:1 and 1:2) were introduced into the colloidal solution under stirring for 1 h. Afterward, the obtained particle suspension underwent a 2-day hydrothermal treatment (70 and 90 °C). Ethanolic hydrochloric acid was used to remove the surfactant templates. Samples were collected by centrifugation and stored in 99.5% ethanol. The synthesis of negatively charged MSNs was identical except for the postmodification with THPMP-silane (2.2 mmol).

### Characteristics of MSNs

Transmission electron microscopic (TEM) images were taken on a Hitachi H-7100 instrument at 75 kV. Sigma Scan Pro 5.0 software (Ashburn, VA, USA) was used for NP size distribution analysis. Dynamic light scattering (DLS) measurements of MSNs suspended in phosphate-buffered saline (PBS) and serum medium (DMEM+10% FBS) were performed on a Nano ZS90 laser particle analyzer (Malvern Instruments, U.K.). Zeta potentials of MSNs were measured in an aqueous solution ranging from pH 6.0 to 8.0. X-ray powder diffraction was measured by X’ Pert PRO (PANalytical) powder using Cu Kα1 radiation (λ = 1.54 Å), and the interplanar spacing was calculated from the Bragg formulation. The N_2_ adsorption–desorption isotherms of the MSNs were obtained from a Micrometrics ASAP 2020 (Norcross, GA, USA). The surface area and pore size were calculated using the Brunauer-Emmet-Teller (BET) equation and the standard Barrett–Joyner–Halenda (BJH) method. A thermogravimetric analysis (TGA) was recorded from 40 to 800 °C on a thermal analyzer with a heating rate of 10 °C/min with an air purge of 40 mL/min. The carbon, nitrogen, oxygen, and hydrogen percentages in the dried sample were measured with an elemental analyzer (Elementar Vario EL cube type for NCSH, Germany).

### *In Vitro* Blood–Brain Barrier (BBB) Model

The *in vitro* BBB model was constructed as previously described.^80,81^ Briefly, human brain endothelial cells were seeded on the bottom side of a transwell insert’s collagen I-coated polycarbonate membrane (0.4-μm pore size; Costar, Corning) at a density of 1.5 × 10^4^ cells/cm^2^. Before the experiment, the BBB culture dish was incubated at 37 °C and 5% CO_2_ for 4 days to reconstruct the tight junctions. The trans-endothelial electrical resistance (TEER) was measured to determine the cell monolayer integrity of the BBB by using an epithelial volt-ohm meter (10 μA current at 12.5 Hz). The medium in the apical side of the BBB model was supplemented with 0.1 mg/mL of RMSN_25_–PEG-TA(2:1), RMSN_25_–PEG-THPMP, RMSN_50_-PEG-TA(2:1), or RMSN_50_-PEG-THPMP and then cultured for 6 h. Furthermore, the culture media on the basolateral side were collected to detect silica concentrations by an inductively coupled plasma optical emission spectroscopic (ICP-OES) analysis. The transport efficiency across the BBB was calculated using the formula shown in the Supporting Information.

For the *in vitro* BBB models of DOX@MSN_25_–PEG-TA transportation, DOX (10 μM) and DOX@MSN_25_–PEG-TA (an equivalent dose of 10 μM DOX) were added following the same procedure as described above. Finally, the culture media in the basolateral side were collected and analyzed by fluorescence spectrometry for the DOX intensity. Based on the linear dependence of the fluorescence intensity on the concentration in the medium over the effective concentration range, the transport efficiency was calculated according to the formula given in Supporting Information.

### Cell Line and Cell Culture

The 4T1 mouse mammary tumor cell lines and U87-LUC human glioma cells (luciferase expressing U87 cells) were cultured in RPMI 1640 (Gibco) and minimum essential medium (MEM, Gibco) supplemented with 10% fetal bovine serum (FBS, Gibco) and 1% penicillin/streptomycin (Hyclone) at 37 °C in a humidified atmosphere containing 5% CO_2_/95% air.

### *In Vivo* Multiphoton Imaging

Seven-week-old healthy ICR mice (BioLASCO, Taiwan) were intravenously injected with 25 nm of RMSN_25_–PEG-TA(2:1) or RMSN_25_–PEG-THPMP at a dose of 200 mg/kg body weight (BW). Real-time images of the blood vessels in the earlobe were taken by multiphoton microscopy (Olympus FVMPE-RS), which was equipped with an IR laser with tunable excitation wavelength ranging from 700 to 1080 nm. The time-lapse images of the circulation of MSNs were captured within 1 to 48 h.

After that, mice were anesthetized, and the procedure of a mouse skull-removal craniotomy was performed according to previous reports.^82−84^ After the craniotomy, mice were placed onto the multiphoton microscopic stage. To visualize the cerebrovasculature, 60 μL of 2.5% (w/v) fluorescein isothiocyanate dextran (FITC-dextran, Mw: 70 kDa) dissolved in sterile saline was intravenously injected through the tail vein for each blood vessel labeling. High-resolution images of MSNs crossing the BBB in the mouse cerebrum were acquired at a depth of 300 μm below the cortical surface (axial spacing: 1 μm) for producing the three-dimensional images.

To evaluate the efficacy of DOX@MSN_25_–PEG-TA for brain tumor targeting and BBB penetration, U87-LUC orthotopic xenograft tumor BALB/c nude mice (7-week-old) were employed. After 20 days, mice were injected with DOX alone (7.5 mg/kg BW) and an equivalent DOX dose of DOX@MSN_25_–PEG-TA via a tail vein for 4 h. Four hours after the injection, mice with a craniotomy were placed onto the multiphoton microscopic stage to visualize DOX localization inside the brain.

### Immunofluorescence (IF) Staining Analysis

After 48 h of treatment with RMSN_25_–PEG-TA(2:1) or RMSN_25_–PEG-THPMP, ICR mice were sacrificed. The brains were excised, fixed in paraformaldehyde (4%, 12 h), and dehydrated by gradient sucrose solutions (10% to 30%), and then and then brain slices were prepared with a frozen section machine. Sections were stained with a primary antibody against CD-31 (1:300) at 4 °C overnight and secondary goat antirabbit IgG-FAM 488 at room temperature for 1 h to visualize the cerebrovasculature (green). Images were obtained with fluorescence microscopy and were analyzed by ImageJ software.

To investigate the mechanisms for crossing the BBB, U87-LUC orthotopic xenograft tumor-bearing BALB/c nude mice were administered with RMSN_25_–PEG-TA (200 mg/kg BW) and were sacrificed after 48h. Then the brains were excised and perfused with paraformaldehyde for the frozen section procedure, and sections were stained with CD-31 (1:100), CD140b (1:100), and zonula occludens (ZO)-1 (1:100) to respectively visualize the cerebrovasculature, pericytes, and tight junction proteins. Images were acquired with a confocal microscope (Leica Stellaris 8).

To evaluate the distribution of MSNs in the brain of U87-LUC orthotopic xenograft tumor-bearing NU/NU mice, RMSN_25_–PEG-TA (200 mg/kg BW) or DOX@RMSN_25_–PEG-TA with a dose of DOX (10 mg/kg BW) was administered every 4 days for three times. Then, mice were sacrificed 24 h after the last injection. Following the above procedure, mice brains were collected and fixed to prepare frozen sections. All nuclei stained with DAPI were used to assess the brain morphology and identify the tumor tissues as regions with hypercellularity. The DOX or RITC-conjugated MSNs were observed by detecting their red fluorescent signals. Finally, images were acquired with fluorescence microscopy (TissueFAX Plus, TissueGnostics, Austria).

### *In Vivo* Biodistribution of MSNs in 4T1 Tumor-Bearing Mice

*In vivo* biodistribution images of RMSN_25_–PEG, RMSN_25_–PEG-TA(2:1), RMSN_25_–PEG-TA(1:2), and RMSN_25_-TA were obtained from a fluorescence imaging instrument (*in vivo* imaging system (IVIS), Lumina). The BALB/c mice (6-week-old) were purchased from BioLASCO (Taiwan) and were subcutaneously implanted with 4T1 tumor cells (2 × 10^6^ cells) to establish a heterotopic allograft model. When the diameter of the tumor reached around 5–10 mm, mice were intravenously injected with various types of MSNs at a dose of 200 mg/kg BW. At 24 h after the injection, mice were euthanized. Major organs (heart, liver, spleen, lungs, and kidneys), tumors, blood, and urine, were excised for imaging, and fluorescence intensity was recorded using an IVIS.

### *In Vivo* Biodistribution of RMSN_25_-PEG-TA in U87 Orthotopic Tumor-Bearing Mice

In vivo biodistribution images of RMSN_25_–PEG-TA(2:1) in mice were captured using a fluorescence imaging instrument (IVIS, Lumina). Seven-week-old BALB/c nude mice, obtained from BioLASCO (Taiwan), were subcutaneously implanted with U87 glioma cells to establish an orthotopic xenograft tumor model. After 2 weeks, mice were intravenously injected with RMSN_25_–PEG-TA(2:1) at a dose of 200 mg/kg. Following a 24-h injection period, the mice were euthanized. After perfusion, the major organs (heart, liver, spleen, lung, kidney, brain, and tumors) were excised for imaging, and fluorescence intensity was recorded using an IVIS imaging system.

### Preparation of DOX-Loaded MSNs (DOX@MSN_25_-PEG-TA)

The DOX-loading procedure was based on previous reports.^85,86^ The MSN_25_–PEG-TA were suspended in a sodium bicarbonate solution (0.1 M, pH 9.95) for 15 min and washed with deionized water before mixing with a DOX aqueous solution for another 1 h. The obtained NPs were denoted as DOX@MSN_25_–PEG-TA, and the drug-loading capacity was determined by measuring the fluorescence spectrum of DOX (excitation ay 480 nm and emission at 590 nm).

### *In Vitro* Drug Release of DOX@MSN_25_-PEG-TA

DOX@MSN_25_–PEG-TA (1.5 mg) were added to a mini-dialysis tube and inserted into an Eppendorf tube containing 1.7 mL of PBS at pH 5.5 and 7.4 under stirring at 37 °C. At selected time intervals, 100 μL of the released solution was taken out to determine the drug concentration, and an equal amount of fresh PBS was added. The drug release profile of DOX@MSN_25_–PEG-TA was determined by measuring the fluorescence spectrum of DOX (excitation at 480 nm and emission at 590 nm).

### Degradation Behavior of MSN_25_-PEG-TA and DOX@MSN_25_-PEG-TA

To explore the degradation behavior of MSN_25_–PEG-TA and DOX@MSN_25_–PEG-TA, the nanoparticles were dispersed in a PBS buffer solution (0.2 mg/mL) and incubated at 37 °C for duration of up to 7 days. The morphology, hydrodynamic size, and count rate of the nanoparticles in the solution were assessed through TEM observation and DLS measurements.

### Cell Viability Assay

U87MG cells were cultured in DMEM supplemented with 10% FBS and 100 μg/mL penicillin/streptomycin at 37 °C in a humidified atmosphere containing 5% CO_2_. The cells were seeded in 96-well plates at a density of 10,000 cells per well and allowed to adhere for 24 h. Subsequently, the cells were treated with 100 μL of various concentrations of DOX@MSN_25_–PEG-TA(2:1), DOX (1, 2.5, 5, 10, 20 μg DOX/mL), or MSN_25_–PEG-TA(2:1) (20, 50, 100, 200, 400 μg MSN/mL). Following a 24-h incubation period, the cell viability of U87MG cells was assessed using the Cell Counting Kit-8 (CCK-8). For the control, cells were maintained in culture medium without any treatment. The absorbance at 450 nm was measured, and the absorbance of the blank solution (100 μL of CCK-8 reagent) was subtracted from both the control and sample readings. All experiments were conducted in triplicate. Cell viability was calculated using the following formula: Cell viability (%) = (A sample - A Blank)/(A control - A Blank) x 100%.

### U87-LUC Orthotopic Xenograft Tumor-Bearing NU/NU Mice

All animal experiments were approved by the Institutional Animal Care and Use Committee (IACUC) of Chang Gung University. Seven-week-old male NU/NU mice were purchased from BioLASCO (Taiwan) and were housed and maintained under pathogen-free conditions at Chang Gung University. Orthotopic brain tumors were established according to our previous experimental procedures.^87^ The skull of a mouse was drilled to create a hole 0.5 mm anterior and 2 mm lateral to the bregma. U87-LUC glioma cells (3 μL of 5 × 10^5^ cells/μL) suspended in MEM were injected at a depth of 2 mm from the brain surface of a mouse by a gastight syringe (Hamilton).

### Magnetic Resonance Imaging (MRI)

Brain tumor growth in U87-LUC orthotopic xenograft tumor-bearing NU/NU mice treated with PBS (control), MSN_25_–PEG-TA (750 mg/kg), DOX (10 mg/kg), or DOX@MSN_25_–PEG-TA (DOX: 10 mg/kg BW) was monitored by MRI. MRI images were acquired on a 7-T magnetic resonance scanner (Bruker ClinScan, Germany). Animals were anesthetized by inhalation of 2% isoflurane following the MRI process,^87,88^ placed in an acrylic holder, and positioned in the center of the magnet. MRI images were acquired using T2-weighted turbo-spin–echo sequences with the following parameters: pulse repetition time (TR)/echo time (TE) 2540/41 ms; FOV = 20 × 30 mm^2^ (156 × 320 pixels); and slice thickness = 0.5 mm. MRI images (transverse and longitudinal slices) of tumor-bearing mice were measured 13 days after tumor implantation, and surviving mice of the DOX@MSN_25_–PEG-TA-treated group were imaged again on day 34.

### *In Vivo* Therapeutic Efficacy of DOX@MSN_25_-PEG-TA

Four days after tumor implantation in U87-LUC orthotopic xenograft tumor-bearing NU/NU mice, DOX alone (10 and 7.5 mg/kg BW), DOX@MSN_25_–PEG-TA (equivalent DOX dose), or MSN_25_–PEG-TA (750 mg/kg BW) was administered through a tail vein injection every 4 days for three times. The growth of the brain tumor was measured and quantified as the intensity of luciferase using an IVIS, and the BW and survival rate were monitored during the experimental period.

### Antitumor Activity in a Spontaneous Brain Tumor Model

A spontaneous brain tumor model was created with transgenic mice (FVB/N strain) as described previously.^46,89,90^ Transgenic mice (*n* = 10) were IV administered DOX (7.5 mg/kg BW) or DOX@MSN_25_–PEG-TA at an equivalent DOX dose three times at 4-day intervals in weeks 8 and 12. To assess overall survival (OS), mice were observed until they spontaneously died. Treatment groups were compared in terms of median survival time (MST, weeks) and the percentage increase in the life span (%ILS). Median survival was the time at which fractional survival equaled 50%:
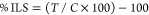
where *T* and *C* are the median survival days of treated and control groups of mice, respectively..

### Single-Dose Toxicity and Histological Analyses

Seven-week-old female BALB/c mice (*n* = 4) were injected with DOX alone (10 and 15 mg/kg BW), DOX@MSN_25_–PEG-TA (equal to DOX: 10 and 15 mg/kg BW), or MSN_25_–PEG-TA (750 mg/kg BW) once via an IV tail vein injection. BWs were recorded on a 14-day schedule. At the end point, the Taipei Medical University laboratory animal center performed blood assays, including a complete blood count (CBC) and blood chemical (BC) analyses. The major organs (heart, liver, spleen, lungs, kidneys, and brain) were fixed with 10% formalin, followed by embedding in paraffin and sectioning to study the toxicological pathology. Then, tissue sections were stained with hematoxylin and eosin (H&E) for a histological analysis. Tissue slides were evaluated by an experienced veterinary pathologist (Toson Technology).

### Pharmacokinetics (PKs) and Quantification of DOX in the Plasma and Brain

Healthy BALB/c mice (7 weeks old, *n* = 3) were given a single dose by IV injection of DOX alone (7.5 mg/kg BW) or DOX@MSN_25_–PEG-TA (equal to DOX: 7.5 mg/kg BW) via a tail vein. To determine DOX concentrations in the plasma and brain, blood samples were taken at 0.25, 0.5, 1, 3, and 24 h after treatment, and each mouse was sacrificed and perfused with PBS for brain collection at selected times. After that, brain tissue extracts (0.4–0.6 g) were homogenized in 600 μL of H_2_O by adding 0.9–1 g homogenized beads (Precellys zirconium oxide beads, 2.8 mm) at 6500 rpm for 1 min twice with a homogenizer (Precellys Evolution, Berlin). DOX from plasma (75 μL) and brain tissues (whole homogenate) was extracted by adding 1 and 3 mL of extract solvent (80% chloroform and 20% methanol) and shaken for 30 min. After centrifugation at 3000 rpm for 10 min, the supernatants were collected and dried in a vacuum. Extraction was repeated three times, and dried supernatants were redissolved in 2 mL of 1.5% hydrogen fluoride (HF) containing DMSO and then sonicated for another 1 h. The concentration of DOX in plasma extracts was measured using a fluorescence microplate reader (SynergyH1 microplate reader, BioTek, with excitation at 480 nm and emission at 680 nm). For brain tissue extraction, the solution was centrifuged at 10^4^ rpm for 30 min three times and filtered through a 0.22-μm PTFE membrane filter to remove the precipitate. DOX concentrations in brain tissue extracts were measured with a fluorescence F-4500 FL spectrophotometer (Hitachi, with excitation at 480 nm and emission at 590 nm).

### Human Plasma

Fresh human blood was collected from both male and female volunteers. The blood was then stored in tubes containing ethylenediaminetetraacetic acid (EDTA) to prevent coagulation. Blood cells and plasma were subsequently separated using centrifugation at a force of 1000 *g* for 10 min, repeated twice to ensure complete removal of all blood cells. Then, the plasma was stored at −80 °C.

### *In Vitro* Protein Corona Formation and Extraction

To form the *in vitro* protein corona, RMSNs were mixed with plasma at a ratio of 1 mg of RMSNs in 50 μL PBS to 300 μL of plasma. This mixture was then incubated for 30 min on a shaker. The PC-NP complex was subsequently separated from human plasma through centrifugation: 1.557 × 10^4^*g* for 30 min for 50 nm RMSNs and 2.0 × 10^4^*g* for 90 min for 25 nm RMSNs. After discarding the unabsorbed plasma proteins, samples were washed with PBS three times for 50 nm RMSNs and four times for 25 nm RMSNs, to ensure complete removal of any unabsorbed plasma proteins. Finally, the collected PC-NPs were resuspended in 20 μL PBS for a subsequent sodium dodecyl sulfate polyacrylamide gel electrophoresis (SDS-PAGE) analysis.

### *In Vivo* Protein Corona Formation and Extraction

Six-week-old ICR mice were purchased from BioLASCO, Taiwan. Mice (*n* = 3 for each NP type) were IV injected with NPs at a concentration of 6 mg/mouse. Following a 5 min interval, mice were anesthetized by inhalation of 2% isoflurane and sacrificed via cardiac puncture. Whole-blood samples were collected in K_2_EDTA tubes, and plasma was isolated by centrifugation (10 min, 900 × *g*). Subsequent washing to remove unbound proteins was performed by a series of five centrifugation steps (1.1 × 10^4^ × *g*, 60 min). The resolved pellet was collected for digestion and mass spectrometry.

### SDS-PAGE

The PC-NP complex was separated using 10% SDS-PAGE, to prepare it for in-gel digestion. To the collected 20 μL of the PC-NP complex, 8 μL of 6× loading buffer (containing 125 mM Tris base, 10% (v/v) glycerol, 2% (wt/vol) SDS, 50 mM DTT, 0.01% (wt/vol) bromophenol blue, and 0.001% (v/v) β-mercaptoethanol) was added. This mixture was then heated to 120 °C for 10–15 min. The NPs and proteins were both subsequently loaded into gel wells. A voltage of 50–60 eV was applied for 2 h to separate the proteins from the NPs. This was followed by 10 min of staining with Coomassie brilliant blue and overnight destaining with water.

### In-Gel Digestion

Corona proteins were isolated from NPs via 10% SDS-PAGE. Resolved bands were stained (Bio-Rad, Coomassie brilliant blue R-250 #161–0436) and excised for digestion. A 1:1 solution of 25 mM ammonium bicarbonate (NH_4_HCO_3_) solution and acetonitrile (ACN) was used to remove the staining solution (3 × , 10 min). Samples were washed with 100 μL ACN followed by reduction via incubation with 100 μL of 10 mM dithiothreitol (DTT) at 60 °C for 1 h. Alkylation was followed by incubation with 60 μL of 55 mM iodoacetamide for 45 min in dark conditions. Next, following further washing with ACN, 80 ng trypsin in 25 mM ammonium bicarbonate was added to digest samples overnight at 37 °C. Samples were subjected to drying and desalting before the mass spectrometric (MS) analysis.

### Liquid Chromatography (LC)–Tandem MS (MS/MS)

The LC-MS/MS analysis was performed on an Orbitrap Fusion Lumos Tribrid quadrupole-ion trap-Orbitrap mass spectrometer (Thermo Fisher Scientific, Bremen, Germany) equipped with a NanoSpray Flex ion source. Peptides were separated on an Ultimate 3000 nanoLC system (Thermo Fisher Scientific) connected to a mass spectrometer. Peptide mixtures were loaded onto a 75-μm-ID, 25 cm-long C18 Acclaim PepMap NanoLC column (Thermo Scientific, San Jose, CA, USA) packed with 2-μm particles with 100-Å pores.

Mobile phase A consisted of 0.1% formic acid in water, and mobile phase B was composed of 100% ACN with 0.1% formic acid. A segmented gradient in 90 min from 2% to 35% of solvent B at a flow rate of 300 nL/min and a column temperature of 35 °C were used. The MS analysis was performed in data-dependent mode with full-MS (externally calibrated to a mass accuracy of <5 ppm and a resolution of 120,000 at *m*/*z* = 200) followed by higher-energy collision-activated dissociation (HCD)-MS/MS of the most intense ions in 3 s. HCD-MS/MS (resolution of 1.5 × 10^4^) was used to fragment multiply charged ions within a 1.4-Da isolation window at a normalized collision energy of 32 eV. AGC targets at 5e5 and 5e4 were respectively set for the MS and MS/MS analyses, with previously selected ions dynamically excluded for 180 s. The maximum injection time was 50 ms.

### ClueGo Pathway Analysis

The ClueGo application1 in Cytoscape was utilized to identify pathways implicated by corona proteins of >0.5% in relative abundance (*n* = 30–50 proteins). Only significant pathways were shown with *p* < 0.05 and a kappa score of 0.68. In ClueGo, the kappa score is used to define term–term interrelations (edges) and functional groups based on shared genes between terms.

## Supporting Information Available

Formula for calculating transport efficiency across the BBB; MSNs characteristics including total surface area, interplanar spacing, and pore size (Table S1); TGA results for MSNs with various modifications (Table S2); Silicon content determination in MSNs using ICP-OES for transport efficiency analysis (Table S3); Characteristics and elemental analysis of MSNs with various modifications (Tables S4, Table S5 and Table S6); TEER values of an in vitro BBB model (Figure S1); Quantitative fluorescence image analysis and IF-stained images of U87 orthotopic mouse brains (Figure S2, S3); Quantitative biodistribution analysis in U87 brain tumor-bearing mice (Figure S4); In vitro degradation of MSNs (Figure S5); Comparative analysis of cell viability in U87MG glioblastoma cells (Figure S6); MRI and histological images of U87 orthotopic mouse brains (Figure S7, S8 and S9); Hematological and biochemical parameters in healthy BALB/c mice (Table S7 and Table S8); Representative histopathologic analysis of major organs in mice (Figure S10); Classification of corona proteins according to molecular weight and isoelectric point (Figure S11) (PDF)
