# Supplementary material for: Receptor Ligand-Free Mesoporous Silica Nanoparticles: A Streamlined Strategy for Targeted Drug Delivery across the Blood–Brain Barrier
Source: ACS Nano. 2024 May 8;18(20):12716–36. doi: 10.1021/acsnano.3c08993 (PMC11112986; doi:10.1021/acsnano.3c08993)
Supplement: Supplementary file 1 — nn3c08993_si_001.pdf [file nn3c08993_si_001.pdf]

Supporting Information

Receptor Ligand-Free Mesoporous Silica Nanoparticles: A Streamlined Strategy for Targeted Drug Delivery Across the Blood-Brain Barrier

Zih-An Chen,[a,b,c†] Cheng-Hsun Wu,[d†] Si-Han Wu,[b,e†*] Chiung-Yin Huang,[f] Chung-Yuan Mou,[a,d] Kuo-Chen Wei,[f,g,h] Yun Yen,[i,j] I-Ting Chien,[a] Sabiha Runa,[a,k] Yi-Ping Chen,[b,e*] Peilin Chen[c*]

[a]Department of Chemistry, National Taiwan University, Taipei 10617, Taiwan
[b]Graduate Institute of Nanomedicine and Medical Engineering, Taipei Medical University, Taipei 11031, Taiwan
[c]Research Center for Applied Sciences, Academia Sinica, Taipei 11529, Taiwan
[d]Nano Targeting & Therapy Biopharma Inc., Taipei 10087, Taiwan
[e]International Ph.D. Program in Biomedical Engineering, Taipei Medical University, Taipei 11031, Taiwan
[f]Neuroscience Research Center, Chang Gung Memorial Hospital, Taoyuan 33305, Taiwan
[g]Department of Neurosurgery, Chang Gung Memorial Hospital, Taoyuan 33305, Taiwan; School of Medicine, Chang Gung University, Taoyuan 33302, Taiwan
[h]Department of Neurosurgery, New Taipei Municipal TuCheng Hospital, New Taipei City 23652, Taiwan
[i]Center for Cancer Translational Research, Tzu Chi University, Hualien 970374, Taiwan
[j]Cancer Center, Taipei Municipal WanFang Hospital, Taipei 116081, Taiwan
[k]SRS Medical Communications, LLC in Cleveland, Ohio 44124, USA.
[†]These authors contributed equally to this work.
Corresponding authors' emails: smilehanwu@tmu.edu.tw; haychen@tmu.edu.tw; peilin@gate.sinica.edu.tw

Experimental Section:

The calculation formula of the transport efficiency across the BBB:

Transport efficiency (%) $= \frac{C_{bottom} - C_{blank}}{C_{upper} - C_{blank}} \times 100\%$;

where C_{upper} is the concentration of silica in the apical side, C_{bottom} is the silica concentration of a transported sample, and C_{blank} is the silica concentration of the medium.

Table S1. Total surface area, interplanar spacing, and pore size of mesoporous silica nanoparticles (MSNs)

Sample	Average size from TEM (nm)	DLS in PBS Z-average (d, nm) / PDI	ζ-potential at pH 7.4 (mV)	d_{100} (nm)	S_{BET} (m^2/g)	D_{BJH} (nm)
$RMSN_{25}$-PEG-TA(2:1)	22.9 ± 2.9	33.2 / 0.09	+4.0	4.14	319.35	1.35
$RMSN_{25}$-PEG-THPMP	21.0 ± 3.3	34.6 / 0.07	−33.8	3.98	428.75	1.50
$RMSN_{50}$-PEG-TA(2:1)	48.1 ± 4.9	55.7 / 0.07	+18	3.85	376.61	1.36
$RMSN_{50}$-PEG-THPMP	46.7 ± 4.6	54.8 / 0.03	−38.2	3.93	589.94	1.72

Z-average, harmonic intensity averaged particle diameter; TEM, transmission electron microscopy; DLS, dynamic light scattering; PDI, polydispersity index; d_{100}, interplanar spacing calculated from the Bragg formulation; S_{BET}, surface area calculated from data using the BET equation; D_{BJH}, pore diameter assigned from the maximum on the BJH pore size distribution.

Table S2. Thermogravimetric analytical (TGA) results for MSNs with various modifications

Sample	TGA results for 40~250 °C (wt%)	TGA results for 250~500 °C (wt%)	TGA results for 500~800 °C (wt%)
$RMSN_{25}$-PEG	17.65%	20.2%	1.92%
$RMSN_{25}$-PEG-TA(2:1)	8.54%	22.9%	2.37%
$RMSN_{25}$-PEG-THPMP	5.23%	25.4%	2.35%
$RMSN_{50}$-PEG	12.10%	14.9%	2.53%
$RMSN_{50}$-PEG-TA(2:1)	17.11%	19.4%	3.23%
$RMSN_{50}$-PEG-THPMP	13.91%	19.4%	2.10%

wt%, normalized weight loss from the TGA.

Table S3. Determination of silicon (Si) by ICP-OES for a transport efficiency analysis

Sample	Initial Si (μg/mL)	Si (μg/mL)	Transport efficiency (%)
$RMSN_{25}$-PEG-TA(2:1)	383.07	20.64	5.4
$RMSN_{25}$-PEG-THPMP	211.84	0.51	0.24
$RMSN_{50}$-PEG-TA(2:1)	204.32	0.45	0.22
$RMSN_{50}$-PEG-THPMP	206.04	0.17	0.08

Initial Si is the initial concentration of silicon in apical medium. Si is the concentration of silicon in basolateral medium. Transport efficiency (%) = Si (μg/mL) / Initial Si (μg/mL).

Table S4. Characteristics of MSNs with various modifications

Sample	Average size from TEM (nm)	DLS in water Z-average (d, nm) / PDI	ζ-potential at pH 7.4 (mV)
$RMSN_{25}$-PEG	30.2 ± 3.6	38.8 / 0.15	-22
$RMSN_{25}$-PEG-TA(2:1)	29.4 ± 3.2	37.5 / 0.13	+4
$RMSN_{25}$-PEG-TA(1:2)	29.1 ± 3.2	37.2 / 0.17	+21
$RMSN_{25}$-TA	29.0 ± 4.4	46.5 / 0.25	+31

TEM, transmission electron microscopy; DLS, dynamic light scattering; PDI, polydispersity index.

Table S5. Elemental analysis of MSNs with various modifications

Samples	Elemental analysis			Ratio of PEG/TA
	C (%)	H (%)	N (%)	
$RMSN_{25}$-PEG-TA(2:1)	18.29	4.49	0.37	2.66
$RMSN_{25}$-PEG-TA(1:2)	16.76	4.55	1.08	0.64

Percentages of carbon, hydrogen, and nitrogen contents for MSNs were obtained from an elemental analysis. PEG, polyethylene glycol; TA, TA-silane.

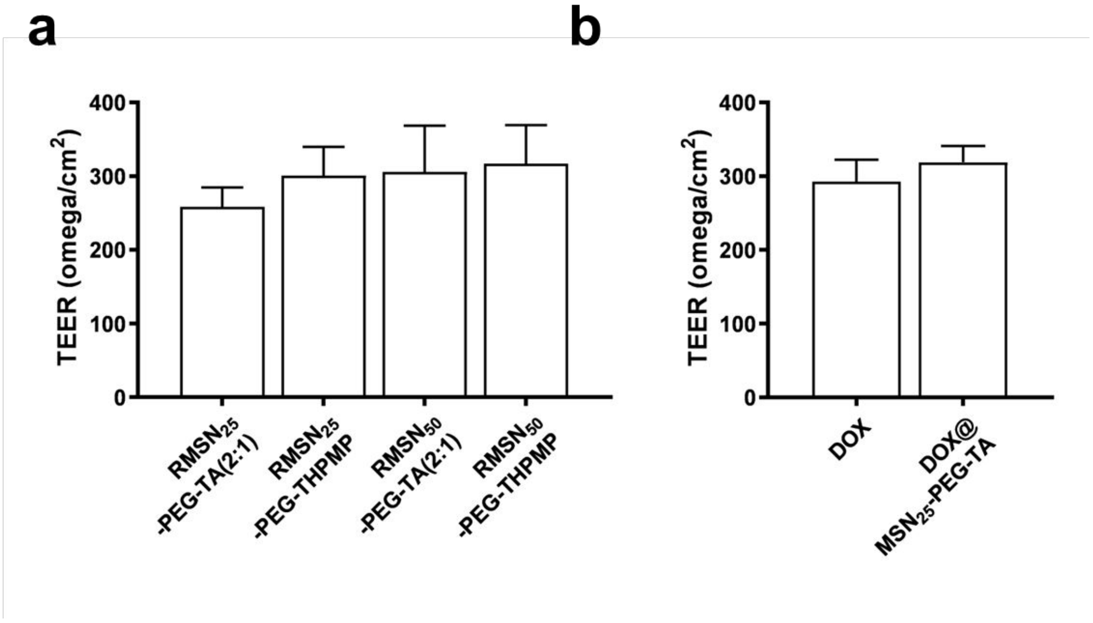

Figure S1. Transepithelial electrical resistance (TEER) values of an *in vitro* blood-brain barrier (BBB) model incubated with (a) 0.1 mg/mL $RMSN_{25}$-PEG-TA(2:1), $RMSN_{25}$-PEG-THPMP, $RMSN_{50}$-PEG-TA(2:1), or $RMSN_{50}$-PEG-THPMP for 6 h. (b) Doxorubicin (DOX; 10 μM) and DOX@MSN_{25}-PEG-TA (an equivalent dose of 10 μM DOX) for 6 h.

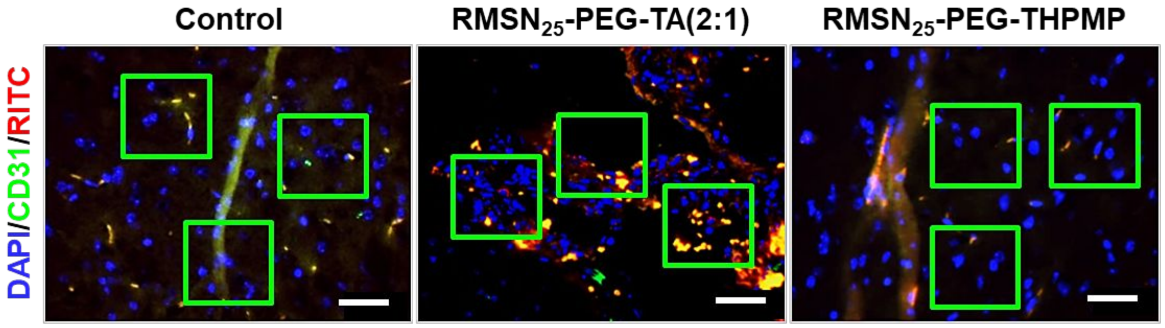

Figure S2. Quantitative fluorescence image analysis based on intensities of regions of interest (ROIs) at three different regions from IF imaging. Scale bar=40 μm.

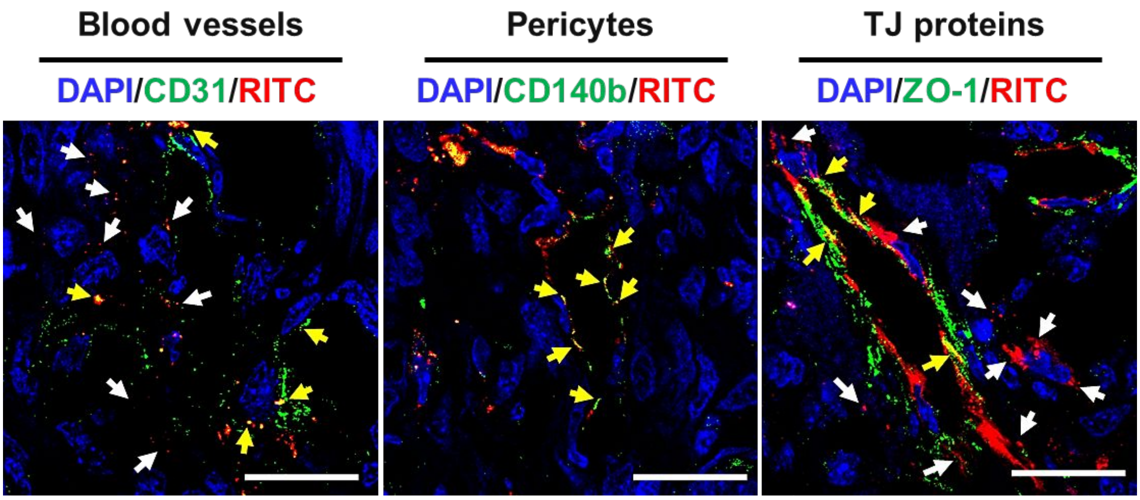

Figure S3. IF-stained images of U87 orthotopic mouse brains. At 48 h post-injection with RMSN$_{25}$-PEG-TA(2:1) at a dose of 200 mg/kg body weight, mice were sacrificed, and frozen sections of the brain were stained with Fluor 488-labeled CD-31 (blood vessels), CD-140b (pericytes), and ZO-1 (tight junctions). Images were captured using confocal microscopy. Red and blue signals represent RITC-conjugated MSNs and DAPI-stained cell nuclei, respectively. Yellow arrowhead: colocalization of red and green signals. White arrowhead: no colocalization of red and green signals. Scale bar=25 µm.

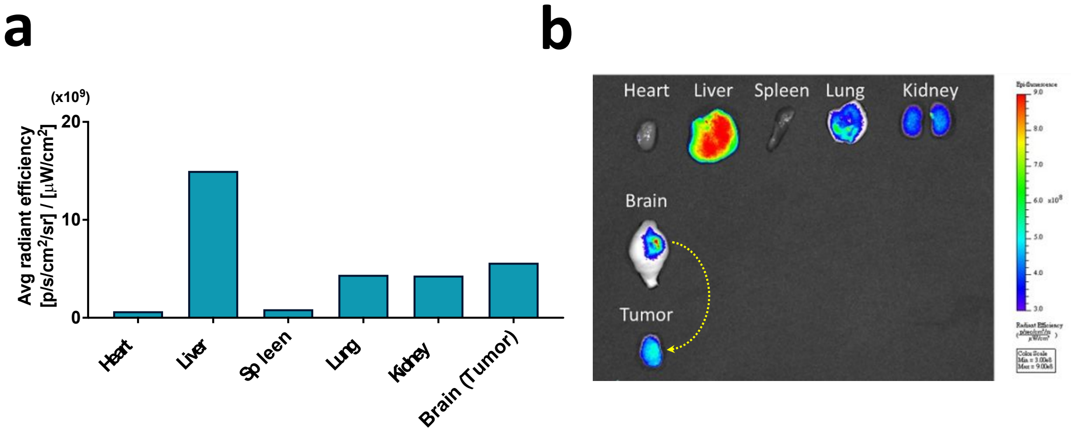

Figure S4. (a) Quantitative biodistribution analysis in U87 brain tumor-bearing mice administered with RMSN$_{25}$-PEG-TA(2:1), as determined by IVIS. (b) Biodistribution images of RMSN$_{25}$-PEG-TA were acquired using the IVIS, with the U87 tumor tissue was removed from the brain.

Table S6. Characteristics of MSN_{25}-PEG-TA and DOX@MSN_{25}-PEG-TA

Sample	D_h in PBS Z-average (d·nm) / PdI	Loading content (LC; %)	Encapsulation efficiency (EE; %)
MSN_{25}-PEG-TA	32.2 / 0.10	-	-
DOX@MSN_{25}-PEG-TA	35.6 / 0.27	3.55%	71%

D_h, hydrodynamic diameter; Z-average, harmonic intensity averaged particle diameter; PDI, polydispersity index; DOX. doxorubicin. LC (%), ratio of the mass of the drug divided by the mass of the nanoparticle; EE (%), mass of the drug encapsulated in nanoparticles divided by the mass of the drug initially present in the solvent mixture.

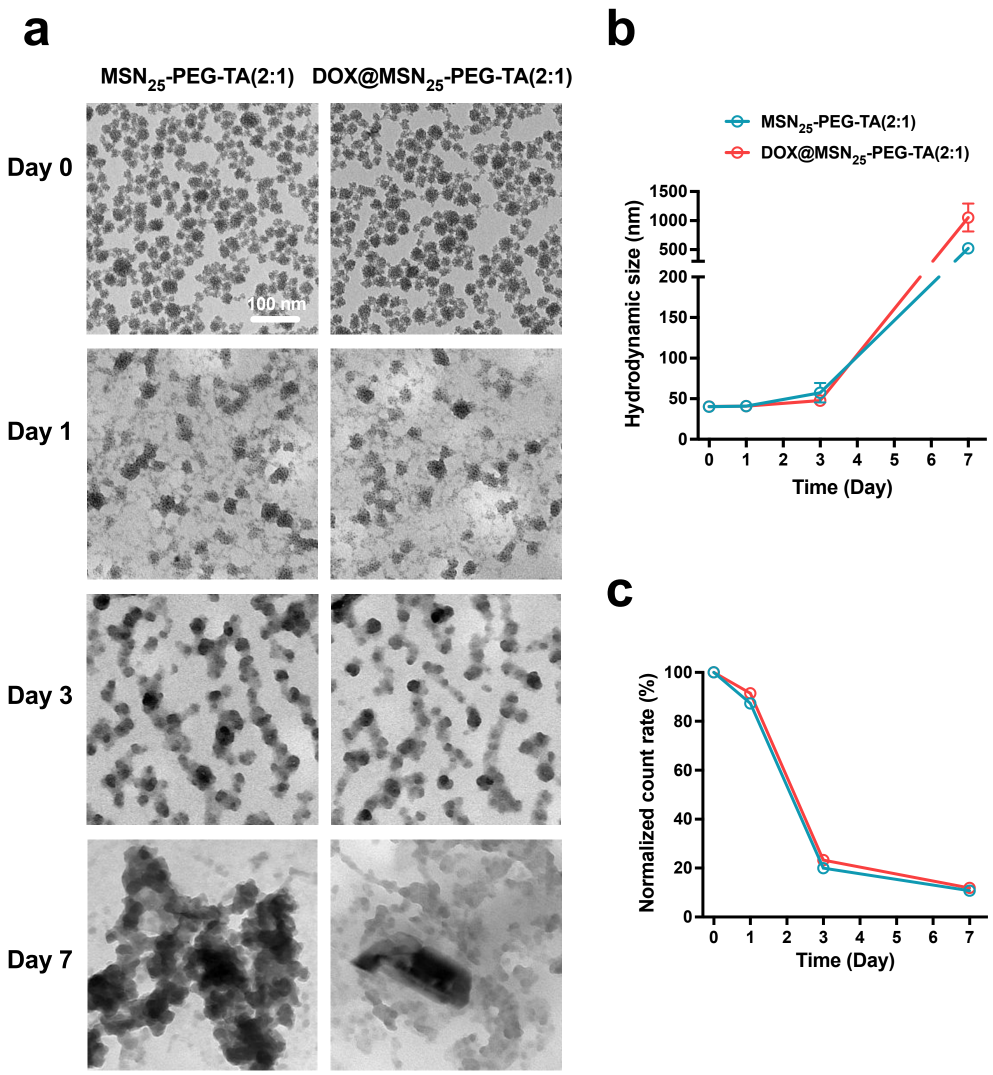

Figure S5. In vitro degradation of MSN_{25}-PEG-TA and DOX@MSN_{25}-PEG-TA, incubated in PBS at 37°C for seven days. (a) The morphology of MSNs in PBS observed by TEM images. (b-c) The size and count rate of MSNs detected by DLS measurement at various time points.

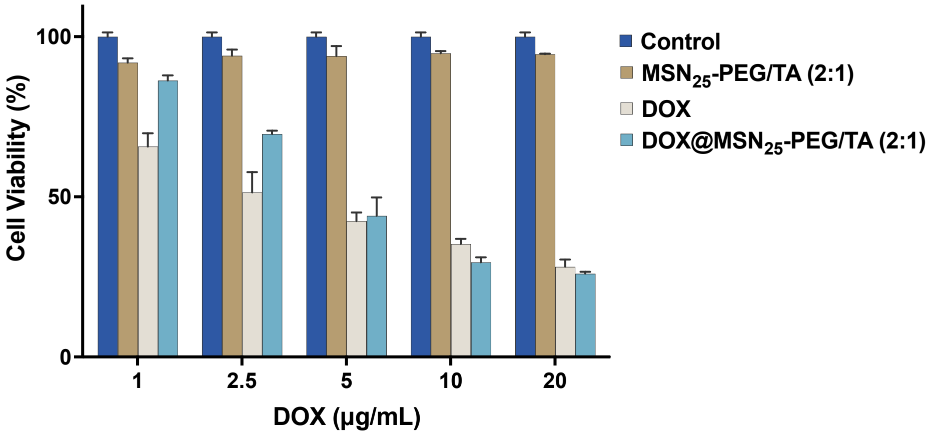

Figure S6. Comparative analysis of cell viability in U87MG glioblastoma cells treated with DOX, MSN_{25}-PEG-TA(2:1), and DOX@MSN_{25}-PEG-TA(2:1).

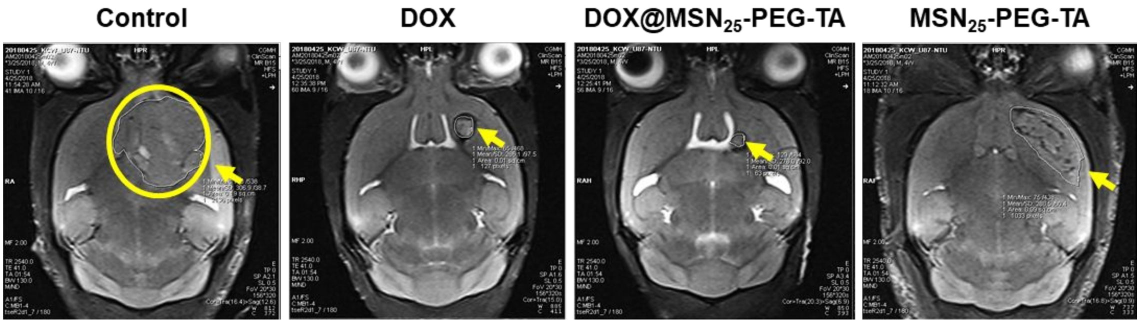

Figure S7. MRI images of U87 orthotopic mouse brains treated with control (PBS), MSN_{25}-PEG-TA (750 mg/kg body weight (BW)), DOX (doxorubicin, 10 mg/kg BW), or DOX@MSN_{25}-PEG-TA [DOX: 10 mg/kg BW] for 13 days. Yellow arrowhead indicates the region of the brain tumor.

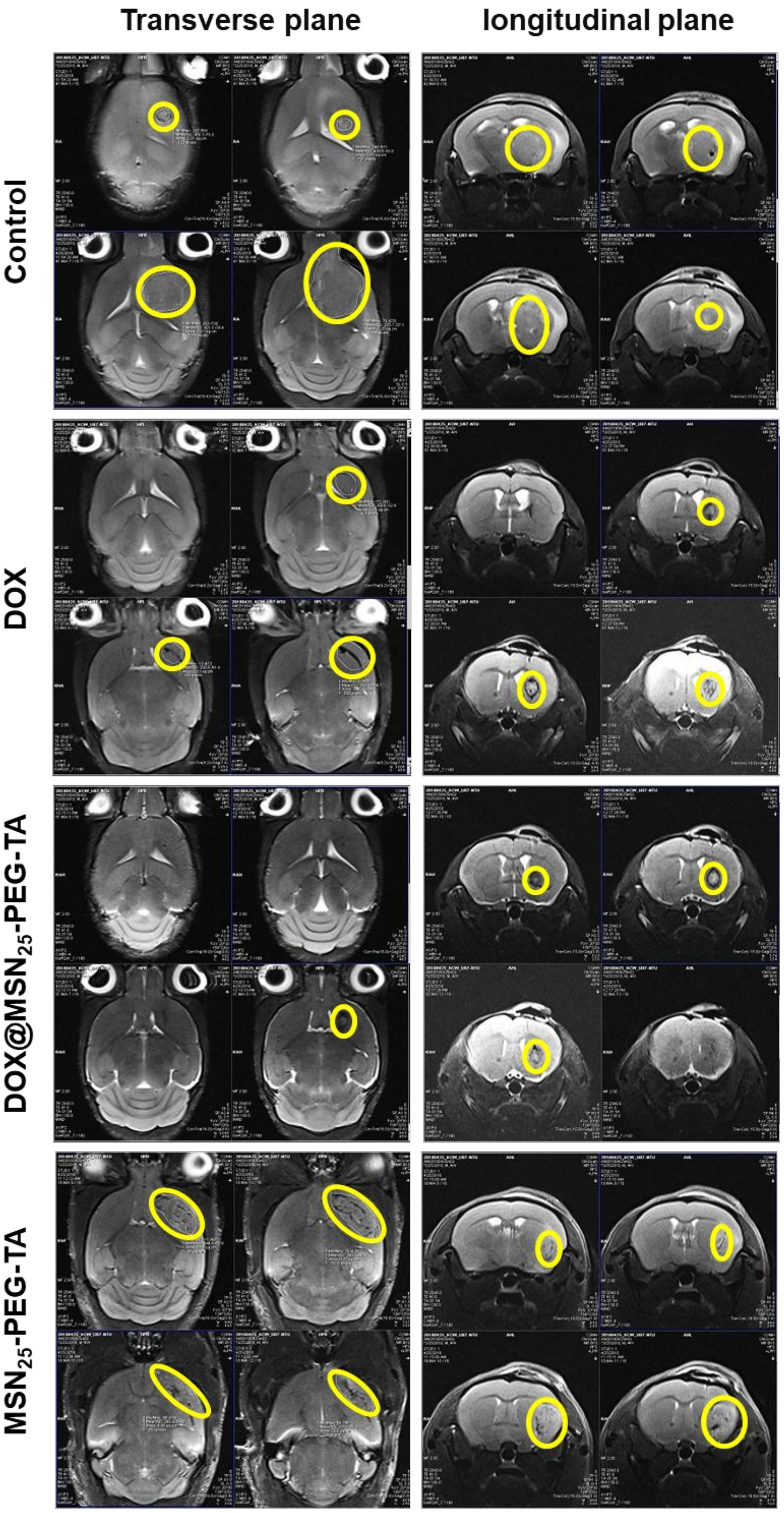

Figure S8. MRI images of transverse and longitudinal slices of U87 orthotopic mouse brains treated with control (PBS), MSN_{25}-PEG-TA (750 mg/kg body weight (BW)), DOX (doxorubicin, 10 mg/kg BW), or DOX@MSN_{25}-PEG-TA [DOX: 10 mg/kg BW] for 13 days. The yellow circle indicates the region of the brain tumor.

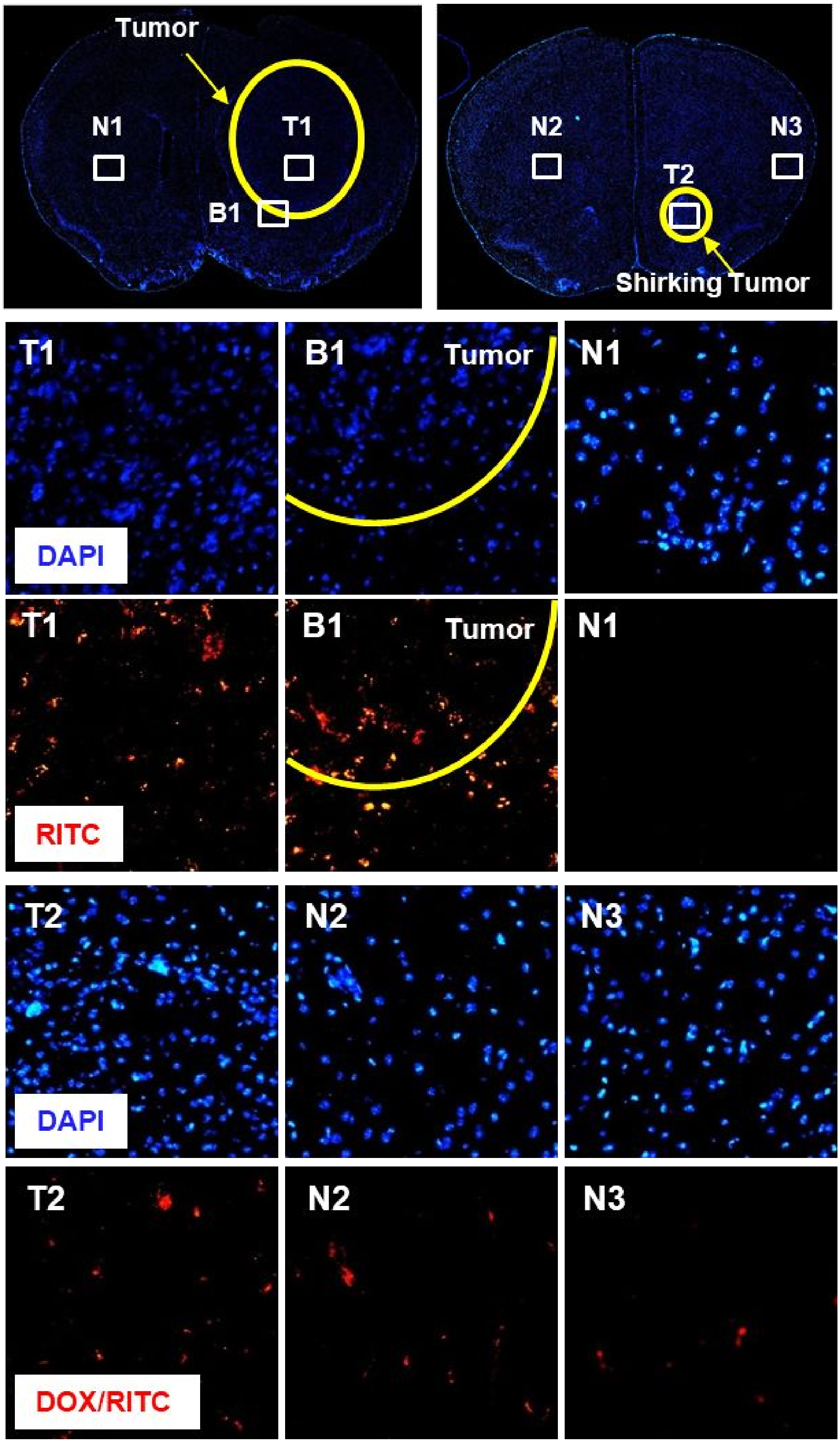

Figure S9. Histological images of the brain in U87 orthotropic xenograft tumor-bearing mice treated with RMSN$_{25}$-PEG-TA (200 mg/kg body weight (BW)) or

DOX@RMSN$_{25}$-PEG-TA at an equivalent dose of DOX (10 mg/kg BW) every 4 days for three times. The red arrowhead indicates the location of the tumor in the brain. The upper left (RMSN$_{25}$-PEG-TA treatment) and upper right images (DOX@RMSN$_{25}$-PEG-TA treatment) show before and present tumor shrinkage, respectively. Blue: nuclei (DAPI). Red: DOX/RITC. Six different regions of interest were selected for further analysis, including locations of the tumor area (T1 and T2), the boundary between the tumor site and normal tissue (B1), and normal brain tissues (N1, N2, and N3). The dotted line in B1 distinguishes the boundary between the tumor site and normal tissues.

Table S7. Values of hematological parameters of the complete blood count in healthy BALB/c mice after control (PBS), DOX (doxorubicin), DOX@MSN$_{25}$-PEG-TA, or MSN$_{25}$-PEG-TA administration

			DOX		DOX@MSN$_{25}$-PEG-TA		MSN$_{25}$-PEG-TA
Test description	Unit	Control	10 mg/kg	15 mg/kg	10 mg/kg	15 mg/kg	750 mg/kg
RBC	(M/μL)	10.94	10.86	9.3*	10.87	11.23	11.2
HGB	(g/dL)	16.63	16.53	14.23*	16.53	16.97	16.85
HCT	(%)	52.67	51.9	44.3*	52.17	53.5	53.85
MCV	(fL)	48.13	47.83	47.6	48	47.67	48.1
MCH	(pg)	15.23	15.2	15.3	15.17	15.1	15.05
MCHC	(g/dL)	31.57	31.83	32.2	31.7	31.7	31.3
RDW-SD	(fL)	32.1	30.53	34.97	31.8	31.43	30.85
RDW-CV	(%)	26.5	25.63	27.6	26.3	26.77	26.05
RET#	(K/μL)	545.73	430.77	607.5*	373.77	465.63	381.75
RET%	(%)	5	3.96	6.47	3.43	4.14	3.41
PLT	(K/μL)	746.67	564.67	737.67	482*	657	727
WBC	(K/μL)	7.72	6.14	7.43	6.34	7.24	7.8
NEUT#	(K/μL)	1.46	1.42	2.81*	1.39	2.32*	2.39
LYMPH#	(K/μL)	6.07	4.4*	4.31*	4.72	4.62	5.23
MONO#	(K/μL)	0.04	0.07	0.15*	0.04	0.08	0.06
EO#	(K/μL)	0.14	0.23	0.15	0.18	0.21	0.13
BASO#	(K/μL)	0.01	0.01	0.01	0	0.01	0.01
NEUT%	(%)	18.73	23.8	37.17*	22.8	31.43*	30.85*
LYMPH%	(%)	78.8	70.77	58.67*	73.57	64.37*	66.75
MONO%	(%)	0.53	1.13*	1.8*	0.6	1.13*	0.7
EO%	(%)	1.77	4.1*	2.2	2.97	2.93	1.65
BASO%	(%)	0.17	0.2	0.17	0.07	0.13	0.05

Abbreviations: *, experimental value differs from the control (red label means above the control; blue label means below the control); RBC, red blood cells; HGB, hemoglobin; HCT, hematocrit; MCV, mean corpuscular volume; MCH, mean corpuscular hemoglobin; MCHC, mean corpuscular hemoglobin concentration; RDW-SD, red blood cell distribution width standard deviation; RDW-CV, red blood cell distribution width expressed as a coefficient of variation; RET, reticulocytes; PLT, platelet count; NEUT, neutrophil count; LYMPH, lymphocyte count; MONO, mononucleosis; EO, eosinophils; BASO, basophils.

Table S8. Values of biochemical parameters in healthy BALB/c mice after control (PBS), DOX (doxorubicin), DOX@MSN_{25}-PEG-TA, or MSN_{25}-PEG-TA administration

			DOX		DOX@MSN_{25}-PEG-TA		MSN_{25}-PEG-TA
**Test description**	**Units**	**Control**	**10 mg/kg**	**15 mg/kg**	**10 mg/kg**	**15 mg/kg**	**750 mg/kg**
BUN	(mg/dL)	25.67	20.67	30.33*	24.67	17.33*	25.33
CREA	(mg/dL)	0.17	0.03*	0.03*	0.1	0.03*	0.03*
TP	(g/dL)	4.73	4.63	4.7	5.2	4.8	5
ALB	(g/dL)	2.03	1.87*	1.73*	2.4	2.3	2.43
ALT	(U/L)	76	85	60.33	62	70.33	62.67
AST	(U/L)	105	77.33	84.33	97.33	92.67	69
ALKP	(U/L)	179.33	115.33	101	128	106.33	185.33
LDH	(U/L)	1614.33	1540.5	2464.67	1670.33	1842.67	1340.67

Abbreviations: *, experimental value differs from the control (red label means above the control; blue label means below the control); BUN, blood urea nitrogen; CREA, creatinine; TP, total protein; ALB, albumin; ALT, alanine transaminase; AST, aspartate transaminase; ALKP, alkaline phosphatase, LDH, lactate dehydrogenase.

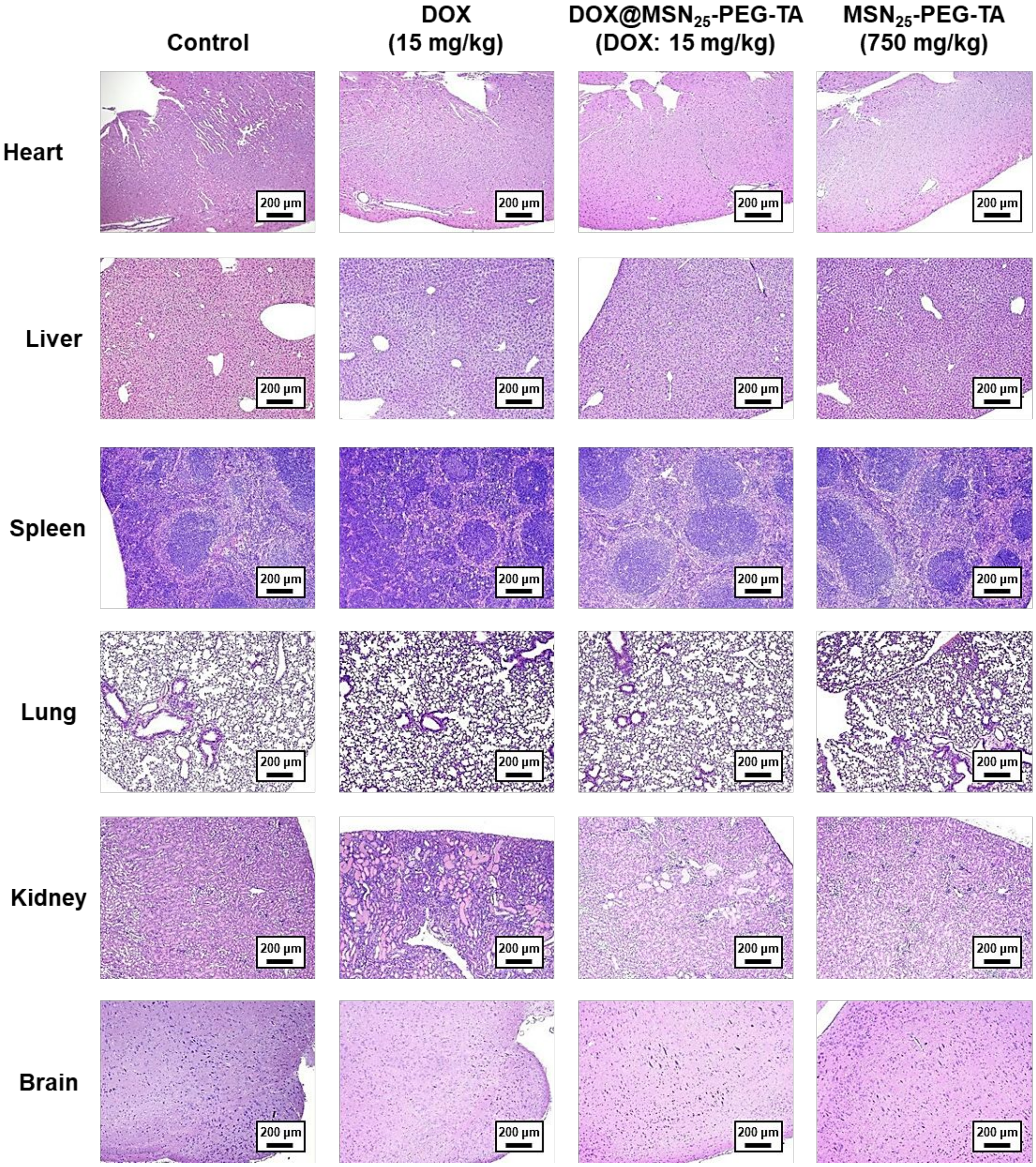

Figure S10. Representative histopathologic analysis of major organs in healthy BALB/c mice with hematoxylin and eosin (H&E) staining. Scale bar=200 μm.

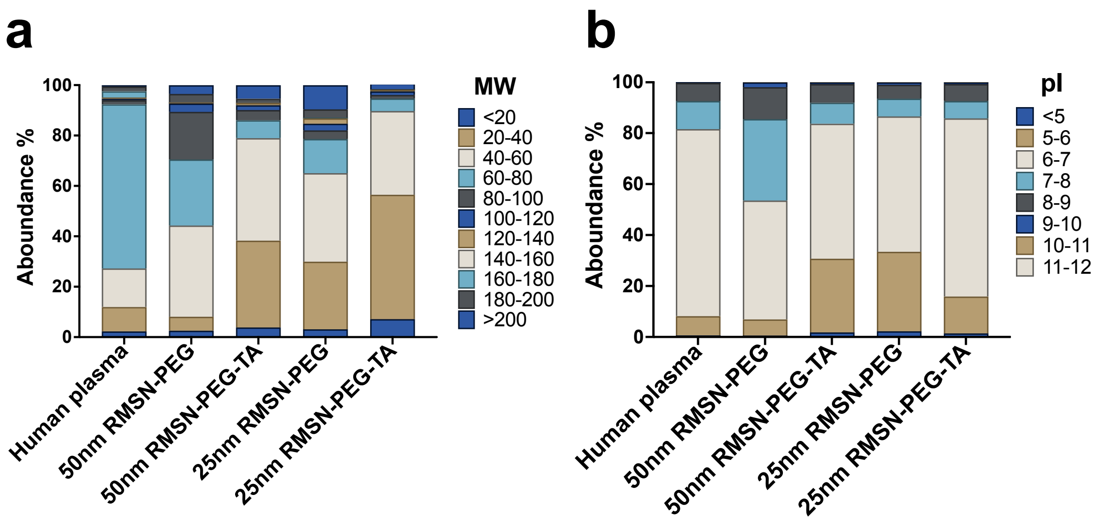

Figure S11. Classification of corona proteins according to their (a) molecular weight (Mw) and isoelectric point (PI) for 50- and 25-nm RMSN-PEG and RMSN-PEG-TA.
